# Deterministic Nucleation of Nanocrystal Superlattices on 2D Perovskites for Light‐Funneling Heterostructures

**DOI:** 10.1002/adma.74290

**Published:** 2026-08-06

**Authors:** Umberto Filippi, Alexander Schleusener, Simone Lauciello, Roman Krahne, Dmitry Baranov, Liberato Manna, Masaru Kuno

**Affiliations:** ^1^ Istituto Italiano di Tecnologia Genova Italy; ^2^ Department of Physics and Astronomy University of Notre Dame Notre Dame USA; ^3^ Division of Chemical Physics and NanoLund, Department of Chemistry Lund University Lund Sweden; ^4^ Department of Chemistry and Biochemistry University of Notre Dame Notre Dame USA

**Keywords:** biexcitons, energy transfer, heterostructures, perovskite nanocrystals, superlattices

## Abstract

Semiconductor heterostructures that combine components with different dimensionalities provide an interesting avenue to manipulate the physical properties of the resulting material. Two‐dimensional lead halide perovskites crystallize as flat microcrystals and have efficient in‐plane exciton mobility, while perovskite nanocrystals are efficient emitters with a tunable bandgap that can self‐assemble into microscopic superlattices. However, combining such intricate architectures into heterostructures has been challenging due to the mismatch in solubility and difficult transfer procedures. Here, we realize heterostructures where CsPbBr_3_ nanocrystal superlattices are deterministically grown along the faces of PEA_2_PbBr_4_ 2D layered perovskite microcrystals. The growth can either be limited to the lateral faces of the microcrystals and result in core–crown epitaxial heterostructures, or extended to the vertical direction leading to core–shell‐like structures. We demonstrate that these heterostructures can be employed as efficient light‐harvesting systems. In fact, energy can be transferred from the 2D microcrystal domain to the superlattices, enabling switching between linear and nonlinear carrier recombination regimes by tuning the excitation fluence. Moreover, by exploiting the lifetime shortening of CsPbBr_3_ nanocrystal emission upon sample cooling, we ensure that energy transfer occurs after the biexcitonic and single‐excitonic decays of the nanocrystals, effectively extending the radiative life time of the superlattices.

## Graphical abstract

This work investigates the heterogeneous nucleation of ${\mathrm{CsPbBr}}_{3}$ nanocrystal superlattices along the faces of 2D ${\mathrm{PEA}}_{2}{\mathrm{PbBr}}_{4}$ layered perovskite microcrystals. Core–crown and core–shell heterostructure morphologies are obtained. Measurements of the time‐resolved emission decays reveal that energy can be transferred from the 2D domain to the superlattice, enabling control of the linear and nonlinear recombination dynamics.

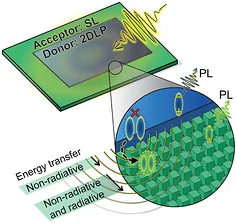

## 1 Introduction

The design of synergistic interactions between different materials is a powerful tool to enhance or transform the properties of individual components that can be accomplished by fabricating heterostructures. Combining different nanomaterials can stabilize one component, boost its performance or even drive the nucleation of a material phase inaccessible by direct synthesis [1, 2, 3].

Two‐dimensional layered metal‐halide perovskites (2DLP) are an interesting class of materials to integrate into heterostructures, since the heterojunction interface can be achieved in the in‐plane or out‐of‐plane directions of the layered stacks, which will have different effects on energy and charge carrier transport [4, 5, 6]. 2DLP‐based heterostructures with bulk perovskites of mixed dimensionalities have also been reported, especially in photovoltaics, where 2DLPs are employed as passivating layers for 3D perovskite crystals to enhance stability [7, 8, 9]. Table 1 surveys the combinations of mixed‐dimensionality perovskite heterostructures reported in the literature.

**TABLE 1:** Perovskite heterostructures with mixed dimensionalities reported in literature.

	3D	2D	1D	0D	NCs
3D	✓ [10, 11]	✓ [12]	✓ [13]	✓ [14]	✓ [15]
2D		✓ [4, 5, 6, 16]	×	×	✓ Isolated NCs [17, 18] ✓ ${\mathrm{Superlattice}}^{\left(\mathrm{This}\mathrm{work}\right)}$
1D			×	×	✓ [19, 20]
0D				×	✓ [21]
NCs					✓ [22, 23, 24]

Heterostructures of mixed dimensionalities incorporating 2DLPs and perovskite nanocrystals have been prepared by epitaxially growing isolated particles atop 2DLP crystals to exploit excitation and charge transfer [17, 18]. An interesting alternative would be to couple 2DLP microcrystals with nanocrystal superlattices, which are 3D ordered solids made of packed and oriented nanoparticles [25]. Such assembly may result in macroscopic structures with enhanced energy and charge transfer between the donor (2DLP) and the acceptor (superlattice). For instance, multiple arrays of adjacent acceptors would allow to exploit the near‐, middle‐ and far‐field coupling regimes, enhancing the overall efficiency of the heterostructure, of which the properties can be finely tuned by engineering the bandgap alignment of the different domains.

The first hurdle to overcome is the heterostructure assembly. Most protocols for superlattice growth rely on slow solvent evaporation or antisolvent‐assisted phase precipitation, which allow nanocrystals to spontaneously aggregate in superstructures [26, 27, 28, 29, 30]. Because of the incompatibility of solubility in a common solvent, nanocrystals cannot be mixed in solution with powders of 2D microcrystals, therefore a post‐assembly strategy is required. Mechanical manipulation could be used to transfer superlattices in proximity of 2DLP microcrystals and fabricate heterostructures, but this tends to generate irreversible deformations due to the softness of the supercrystals [28, 31]. Protocols for the templated growth of particle assemblies have been reported, but they either rely on cumbersome substrate treatments [32, 33], or require nanocrystals to merge irreversibly into superstructures [34].

In this work, we report a successful approach to fabricate heterostructures made of ${\mathrm{CsPbBr}}_{3}$ nanocrystal superlattices and PEA_2_PbBr_4_ 2DLP microcrystals. We use 2DLPs as seeds for the heterogeneous nucleation of nanocrystal assemblies during a slow solvent evaporation. Tuning the evaporation time and the nanocrystal concentration results in heterostructures with core–crown and core–shell morphologies.

**SCHEME 1:**
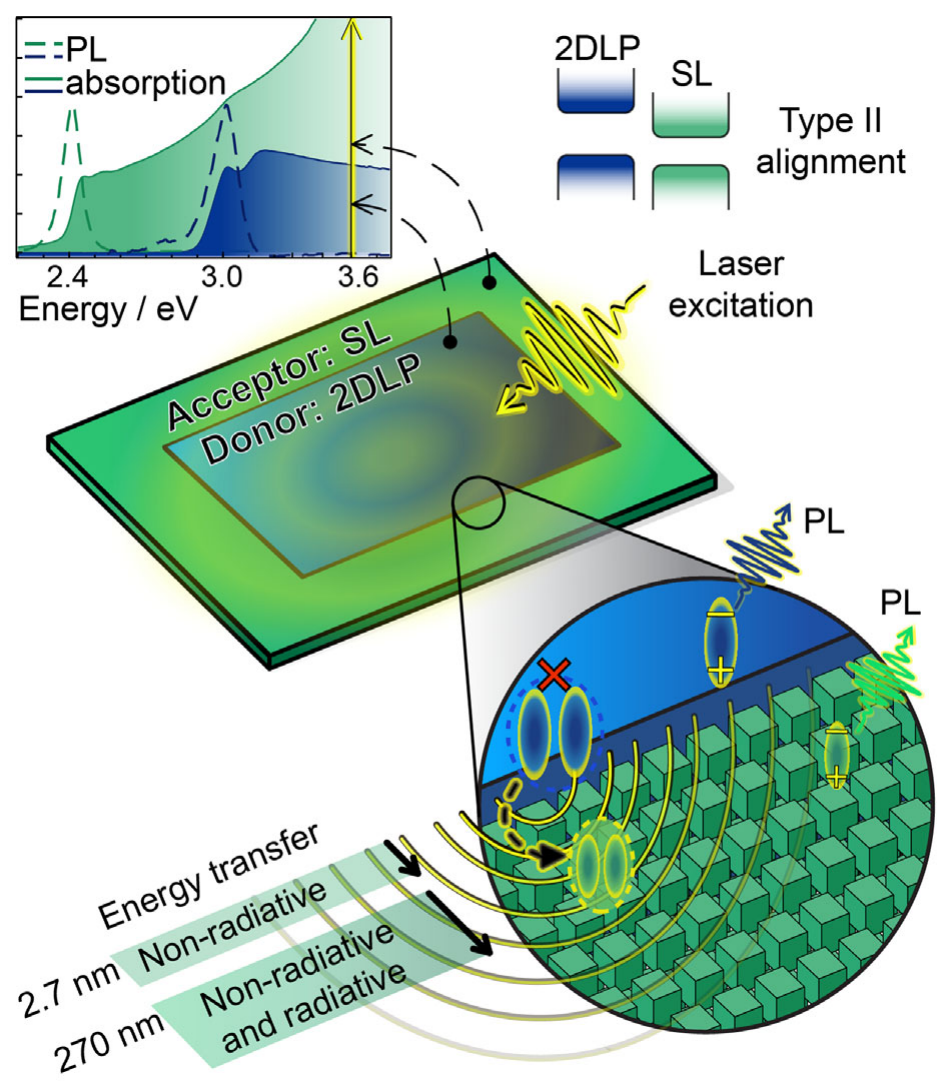
Cartoon summarizing core–crown heterostructures band alignment and optical interaction. After laser excitation, the 2DLP (donor) transfers (non‐radiatively and radiatively) the excitation to the superlattice (acceptor).

We demonstrate the efficient energy transfer from the 2DLP domain to the superlattice, the latter acting as an energy acceptor and radiative component of the system. The efficiency of the energy transfer stems from the exceptionally large Förster radius (≈67 nm), which enables non‐radiative and radiative transfers in the near‐, middle‐, and far‐field domains, and from the core–shell or core–crown configuration of the heterostructures, where a high bandgap material is surrounded by a superlattice of nanocrystals that feature a smaller bandgap (see Scheme 1).

**FIGURE 1:**
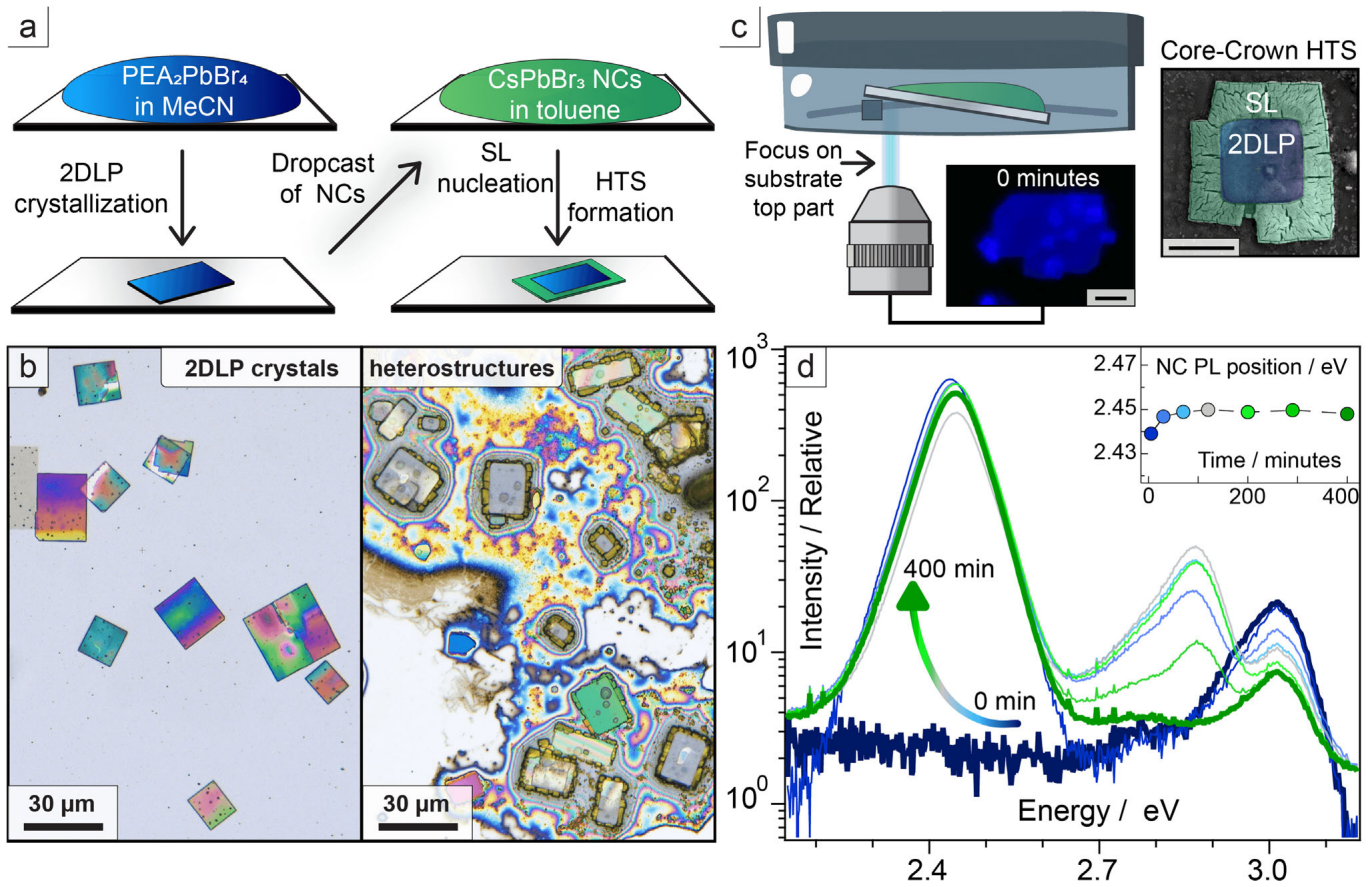
Heterostructure nucleation. (a) Formation of 3D CsPbBr_3_ NC SL/2D PEA_2_PbBr_4_ crystal heterostructures. 2DLP microcrystals are grown on a designated substrate by an antisolvent‐assisted fast crystallization procedure (≈4 h) [4]. Then, a solution of nanocrystals is dropcast onto the same substrate, which is subsequently enclosed in a Petri dish to ensure slow solvent evaporation. After ≈6 h, heterostructures are formed. (b) Representative microscope images of regions of the substrate before (left) and after (right) core–crown heterostructure formation. (c) Scheme of the inverted microscope set‐up used to optically monitor the heterostructure formation (scale bar: 15 μm). On the right a colored SEM image of a typical core–crown heterostructure formed on the substrate top part is displayed (scale bar: 15 μm). (d) In situ PL spectra acquired during heterostructure formation. Inset: nanocrystal PL peak position during heterostructure growth.

**FIGURE 2:**
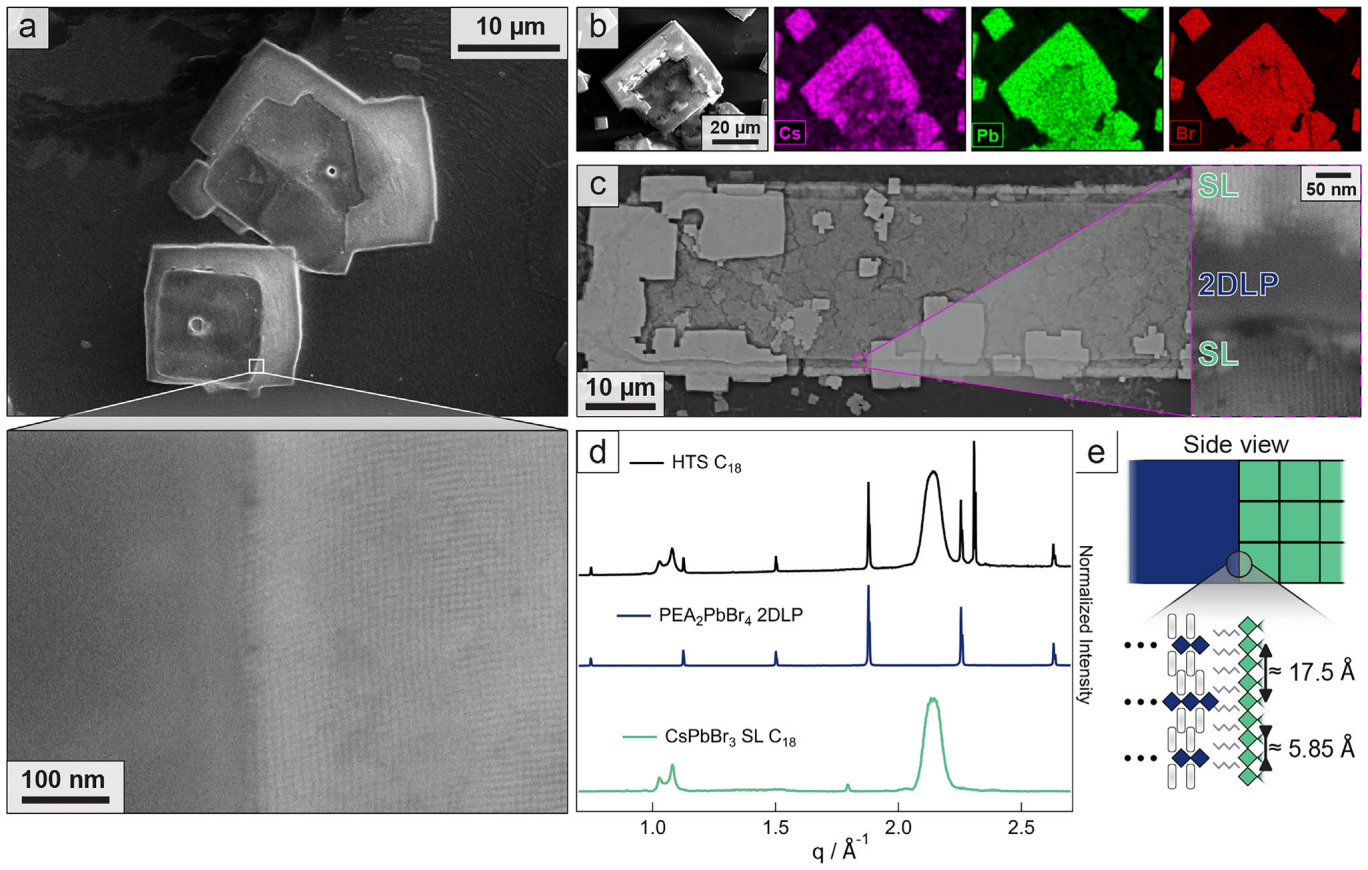
Structural characterization of heterostructures. (a) Top: Low‐magnification SEM images of the heterostructures. 2DLP microcrystals are located at the center of the structures, while superlattices grow at the edges. Bottom: high‐resolution image of a representative heterostructure interface, where arrays of nanocrystals are observed. (b) SEM image of one heterostructure and corresponding EDX maps for Br, Cs, and Pb. (c) Left: Low‐magnification SEM image of a heterostructure with a fully covered 2DLP microcrystal (nanocrystals also assemble on the top surface). Right: High‐resolution image of a representative region of the heterostructure interface. (d) Comparison between ensemble XRD patterns of pure C_18_‐capped CsPbBr_3_ superlattices, pristine PEA_2_PbBr_4_ microcrystals, and PEA‐C_18_ heterostructures (black trace on top). The strong reflection at 2.32 Å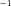, visible in the heterostructure pattern, originates from the silicon substrate [44]. (e) Cartoon depicting a possible crystallographic alignment between 2DLP and nanocrystals.

**FIGURE 3:**
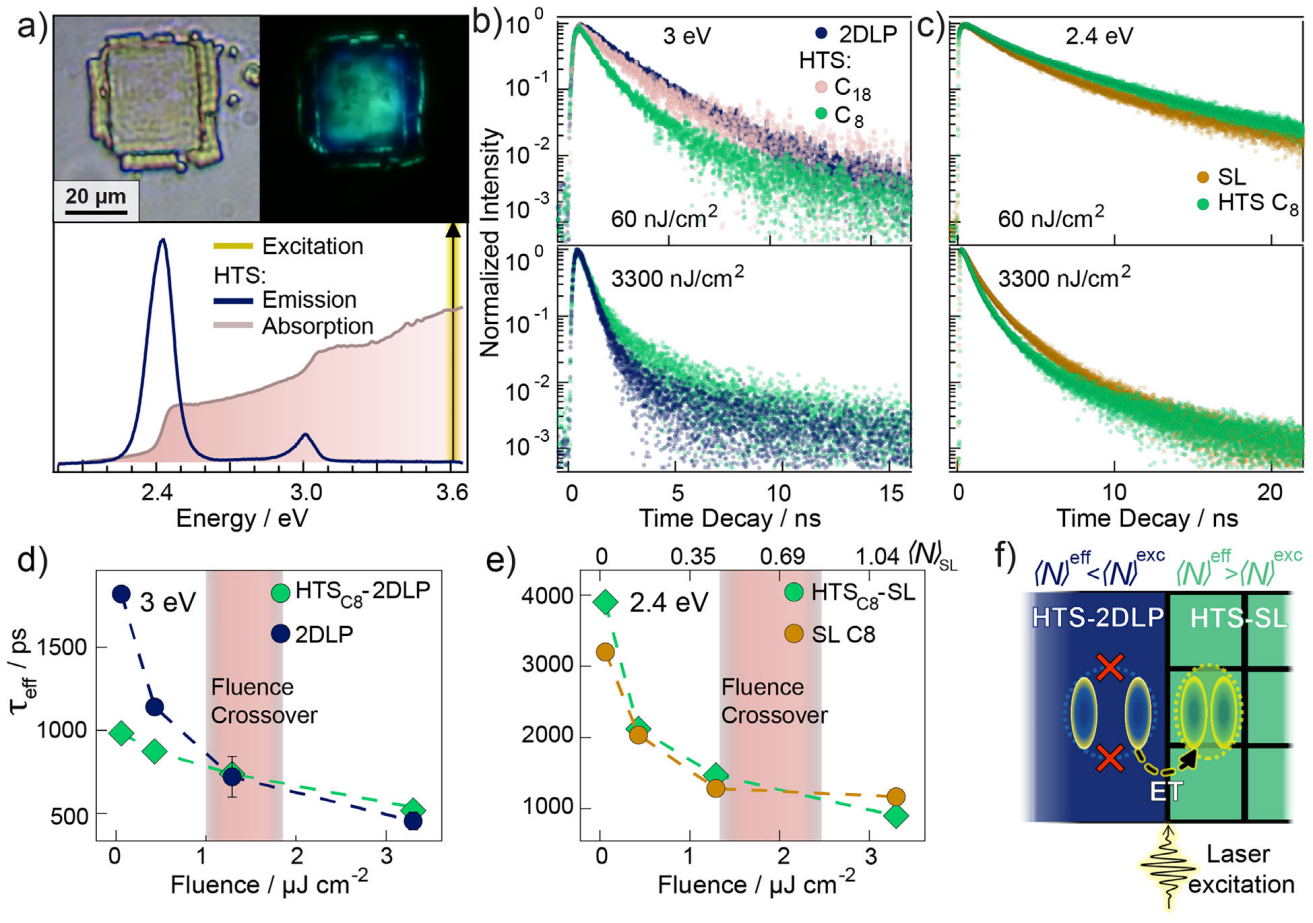
Fluence‐dependent energy transfer. (a) Top: Representative microscope images of one heterostructure excited with (left) visible and (right) UV light. Bottom: Absorption spectrum of several heterostructure ensembles and representative PL spectrum of a single heterostructure. (b) Time‐resolved emission decays of a pristine 2DLP microcrystal and a heterostructure extracted at 3 eV, and collected in the low‐ (top) and high‐fluence (bottom) regimes. (c) Similar comparison for traces extracted at the CsPbBr_3_ nanocrystal emission peak (≈ 2.4 eV) for a pristine $C_{8}$ superlattice (orange line) and a $C_{8}$ heterostructure (spring green line). (d, e) Effective lifetimes (weighted averages from biexponential fits) as a function of fluence, extracted at 3 and 2.4 eV emission energies, respectively. The underlying pink regions indicate the fluence ranges where energy transfer starts to significantly impact biexcitonic recombination relative to single excitonic decays. (f) Cartoon depicting the influence of energy transfer on the average exciton population per recombination site ⟨N⟩.

**FIGURE 4:**
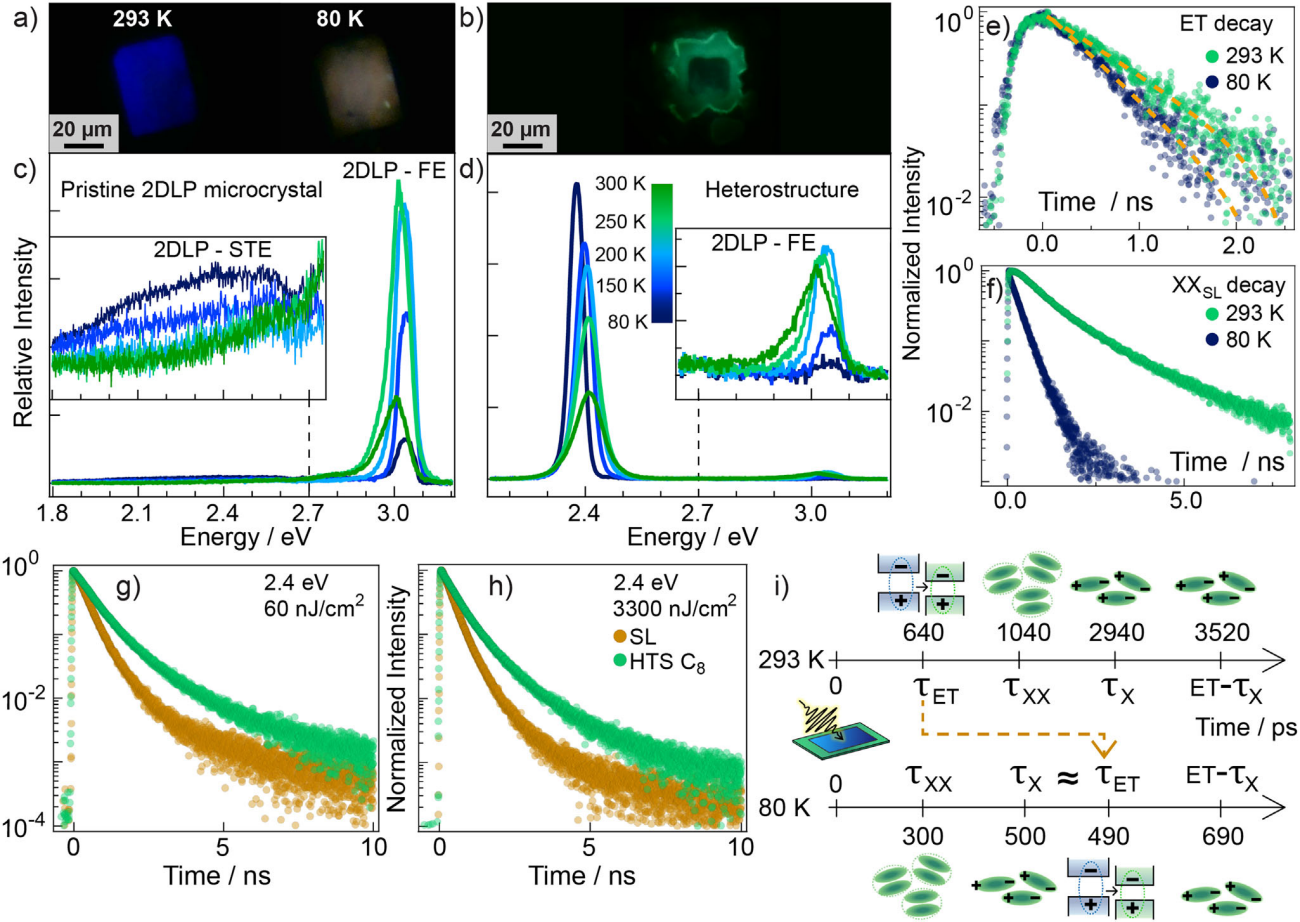
Low‐temperature acceptor radiative enhancement. (a,b) Representative microscope images of a pristine 2DLP microcrystal at 293 K (left) and 80 K (right) (a) and of a heterostructure (b), both excited with a 343 nm laser. (c,d) Temperature‐dependent emission spectra of pristine 2DLP microcrystal and C_8_ heterostructure, respectively. In (c) the inset shows the emergence of the STE at 150 K and in (d) the inset highlights the temperature evolution of the 2DLP peak. (e) Energy transfer decays extracted at 80 K and 293 K from the 2DLP emission (see Figure S12). The orange dashed lines correspond to the best single‐exponential fits. (f) Biexcitonic decays extracted from the superlattice emission at 293 and 80 K (see Figure S16). (g,h) Time‐resolved emission of a heterostructure and of a pristine superlattice extracted at the CsPbBr_3_ emission peak in the low‐ (g) and high‐fluence (h) regimes. (i) Scheme of all the excitation and recombination mechanisms occurring in the superlattice domain of the heterostructure at 293 K and 80 K.

Moreover, we achieve energy transfer‐mediated control over the nonlinear phenomena in heterostructures, as the donor domain exhibits a partial suppression of biexciton recombinations due to the depopulation caused by energy transfer. As for the acceptor, we identify different temperature regimes to tailor the recombination timescales with respect to the energy transfer, which allows to either accelerate non‐radiative biexciton decays or to extend the lifetime of radiative single excitons.

Overall, we demonstrate the capability to assemble powerful light‐harvesting systems with switchable optical properties. The remarkable harvesting efficiency is directly linked to the large light‐absorption cross section of the individual building blocks, whereas the transfer efficiency arises from the strong spectral overlap and geometric design of the heterostructures (Scheme 1), which are characterized by complete or quasi‐complete coverage of the donor domain. The combination of these effects transforms the 2DLP microcrystal into an efficient energy funnel that transfers the laser excitation into the superlattices. Further advancements could lead to the development of bio‐inspired heterostructures sensitive to extremely low light conditions and capable of funneling the excitation into designated reaction centers.

## 2 Results and Discussion

### 2.1 Heterostructure Formation

The preparation of heterostructures is summarized schematically in Figure 1a and consists of two separate steps: the growth of large n=1 PEA_2_PbBr_4_ 2DLP microcrystals (see Figure S1) [4, 35, 36], and the subsequent growth of superlattices by slow solvent evaporation after drop‐casting the nanocrystal dispersion. We used toluene dispersions of CsPbBr_3_ nanocrystals passivated with mixed ligands (oleic acid and oleylamine or octylamine, further referred to as $C_{18}$ and $C_{8}$, respectively, see Figure 1b and Methods) [37, 38]. Heterostructure formation occurs on a tilted substrate, which provides a gradient of drying nanocrystal dispersion (the liquid gets progressively concentrated toward the bottom of the film and hence extends the evaporation time in that region, while drying is faster in the upper part, see Figure 1c). The tilted substrate enables control over the morphology of the resulting heterostructures, as discussed further in the text. For optimized reproducibility, a 3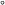 tilt angle was employed (see experimental section). Smaller angles can still lead to heterostructure formation, although with reduced reproducibility due to less controlled solvent flow. Larger angles were instead avoided as they increase the probability of solvent overflow from the substrate.

Regarding the preferential nucleation of superlattices at the edges or on top of the 2DLP microcrystals, we speculate that this is mostly driven by the reduction of the surface energy contribution to the Gibbs free energy. Additional contributions may arise from the local accumulation of nanocrystals during solvent evaporation, which creates supersaturated domains that favor superlattice formation, as well as from van der Waals interactions between the nanocrystals and the 2DLP microcrystal edges.

Heterostructure formation was tracked by monitoring the light emission of the constituent materials, starting from individual 2DLP microcrystals located at the top of the substrate (Figure 1d). The intensity of the 3 eV emission peak of the PEA_2_PbBr_4_ microcrystal decreases immediately after dropcasting the nanocrystal solution, pointing to optical coupling between the two species.

The PL peak of CsPbBr_3_ nanocrystals undergoes a 10 meV blueshift during heterostructure growth, a behavior not observed during growth of pristine superlattices [26]. According to PL sizing curves, this shift corresponds to a nanocrystal size reduction of $\Delta {\mathrm{NC}}_{\mathrm{size}}$ ≈ 0.60 nm, which is approximately one CsPbBr_3_ unit cell length [39, 40]. Simultaneously with that change, a new PL peak appears at approximately 2.870 eV, interpreted as a formation of quasi‐2D PEA_2_CsPb_2_Br_7_ that could be caused by residual ${\mathrm{Cs}}^{+}$ cations transfer to the 2DLP domain [41]. The disappearance of the 2.870 eV PL peak at the end of the growth process indicates that this quasi‐2D PEA_2_CsPb_2_Br_7_ phase does not form a stable interface neither with the nanocrystals nor the 2DLP domains. The growth process described above is representative for heterostructures growing on the top part of the substrate, where the evaporation time is shorter and the nanocrystal concentration is lower. In contrast, heterostructures forming at the center of the substrate more frequently show a layer of nanocrystals on top of the 2DLP and retain the PL peak from the mixed phase components even after growth is completed (Figure S2). Complete dissolution of the 2DLP microcrystals can be observed at the bottom of the substrate (Figure S3), where the long evaporation time enables prolonged dissolution and recrystallization processes. The location on the substrate where heterostructures grow also affects the changes in the emission peak intensity of CsPbBr_3_ nanocrystals, as it influences the number of emitting nanocrystals and their density (see Figures 1d and S2).

### 2.2 Interface Characterization

The ex situ morphology of the heterostructures was characterized by scanning electron microscopy (SEM) and X‐ray diffraction (XRD) measurements. Figure 2a shows the case of core–crown heterostructures, where CsPbBr_3_ nanocrystals assemble with their in‐plane facets aligned to the lateral edges of the 2DLP (inset in Figure 2a). Elemental distribution maps confirm that cesium is predominantly localized in the heterostructure regions occupied by superlattices (Figure 2b, see also Figure S4 for elemental analysis at the cross section). As discussed earlier, the morphology of the heterostructures can be tuned by extending the evaporation time of the nanocrystal solution, allowing nanocrystals to also grow on the top surface of the 2DLP microcrystals (Figure 2c, see also Figure S5). Vertical alignment between superlattices and 2DLP microcrystals is likely to occur, as the two components share a similar corner‐shared (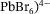 octahedra periodic structure, which may facilitate structural alignment at the interface. Indeed, previous works reported individual nanocrystals exchanging aliphatic ligands with phenethylamine from the 2D component and growing vertically and epitaxially from the top face of the 2D material [17, 18]. In contrast, lateral alignment of the lattices of the two materials is less intuitive due to the mismatch in unit cell periodicities between the two domains. The interlayer spacing d of the (001) planes of the PEA_2_PbBr_4_ microcrystal, calculated from the XRD peak separation as $\Delta q=\frac{1}{d}$, is equal to 16.7 Å (Figure 2d). The first Bragg peak of CsPbBr_3_ centered at 1.05 Å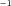 shows the characteristic splitting of $C_{18}$‐capped superlattices [42, 43], and the measured (001) lattice parameter is approximately 5.85 Å (see Figure 2e). Therefore, a possible alignment scenario is that an interface with limited strain can form as three unit cells of CsPbBr_3_ nanocrystals (≈17.5 Å) match the interlayer distance of the PEA_2_PbBr_4_ microcrystal. An alternative explanation arises from analyzing the heterostructure crystallization process. As discussed in the previous section, the growth involves the partial dissolution of the 2DLP edges (likely driven by the free ligands present in the nanocrystal solution), and Cs ion migration from CsPbBr_3_ nanocrystals within the n=1 2DLP edges, leading to the formation of mixed‐phase domains with n>1 (see also Figure S6). These domains generate a mismatch in the periodicity, hence they can easily detach from the main crystal body. These two phenomena could give rise to ladder‐like edges accommodating the nanocrystals both vertically and laterally, which is supported by SEM images taken from the interface of some heterostructures, where alternating regions of nanocrystals and residual 2DLP sheets are observable (Figure S7).

These findings indicate a dynamic reactivity between the 2DLPs and the nanocrystals, involving the exchange of chemical species and resulting in the formation of multiple heterostructure morphologies with different structural properties (see also Figures S8, S9, and S10). It is important to specify that all the possible interfaces discussed are nonclassical, hence they do not imply atomic alignment at the interface, as the lattice matching is mediated by organic ligands.

It is worth mentioning that the 2DLP and the CsPbBr_3_ nanocrystals were prepared with different ligand species, and yet they were able to form organized structures. This underpins the versatility of our assembly method and suggests its potential extension to different materials and their combinations. In the following sections, we focus on the investigation of heterostructures by optical spectroscopy to understand the photophysics of both 2DLP and nanocrystal components and their coupling dynamics.

### 2.3 Energy Transfer at Low Fluence

The literature‐reported type II band alignment of CsPbBr_3_ and PEA_2_PbBr_4_ [17, 45, 46], and the large spectral overlap of the 2DLP microcrystal absorption and nanocrystal PL (see Scheme 1), make these heterostructures a suitable platform for investigating energy and charge transfer processes.

Moreover, the core–shell/core–crown heterostructure geometry combined with the layered architecture of the 2DLP in the center should favor energy transfer: excitons that are created within the 2DLP microcrystal can efficiently diffuse to the edges through the in‐plane inorganic layers, where they may be transferred to adjacent superlattices [47, 48]. In addition, the 2DLP emission can be guided laterally toward the edges of the microcrystal and reabsorbed by the NC superlattices, as 2DLP microcrystals can act as waveguiding slabs. These processes can be further enhanced by edge states of the microcrystals and their emission [49].

Such energy transport and accumulation along macroscopic distances targeting specific recombination centers with nanoscale precision (e.g., the acceptor nanocrystals) could offer a way to potentially control nonlinear optical responses (e.g., by tuning the excitation fluence). However, toward this goal, it is fundamental to investigate the timescales on which the different processes occur.

We therefore employed a time‐correlated single‐photon counting system to measure the time‐resolved emission of individual heterostructures upon exciting them with a 343 nm femtosecond pulsed laser. To focus, a 10× objective lens was employed, resulting in an excitation spot of ≈9.5 μm in diameter. Both 2DLP and superlattice domains were directly excited by the laser beam for the time‐resolved emission studies. Representative microscope images of one heterostructure and its emission and absorption spectra are shown in Figure 3a.

To properly address the changes in the optical properties of the heterostructure components, it is essential to characterize them individually. In this regard, we measured the time‐resolved emission of pristine 2DLP microcrystals and superlattices in the low fluence regime, where their recombination is mainly single excitonic (60 nJ cm^−2^, see top graphs in Figure 3b,c for representative decays). The lifetimes extracted from single exponential fits are found to be $\tau_{X,2\mathrm{DLP}}=1870\pm 200$ ps and $\tau_{X,\mathrm{SL}-C_{8}}=2940\pm 410$ ps for pristine 2DLP microcrystals and superlattices, respectively (the latter assembled from $C_{8}$‐capped nanocrystals, see Figure S11 for the characterization of $C_{18}$‐capped superlattice and heterostructure). When assembled in heterostructures (HTS‐$C_{8}$), the lifetime of the 2DLP domains shortens ($\tau_{{X,\mathrm{HTS}-C}_{8},2\mathrm{DLP}}=830\pm 170$ ps), while the one of superlattice extends ($\tau_{{X,\mathrm{HTS}-C}_{8},\mathrm{SL}}=3520\pm 570$ ps). Both phenomena indicate that energy is transferred from the 2DLP to the nanocrystals, as commonly observed in donor–acceptor systems [50, 51, 52].

Within the framework of Förster resonance energy transfer (FRET), the rate of transfer is 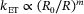, where $R_{0}$ is the Förster radius, which represents the distance at which energy transfer and donor emission can occur with the same probability, R is the donor–acceptor distance and m depends on the transfer zone (i.e., m=6,4,2 for near, intermediate‐ and far‐field zone respectively) [53, 54, 55]. Because energy transfer includes both non‐radiative and radiative contributions that have different scalings, depending on transfer zone, computing $k_{\mathrm{ET}}$ analytically is particularly challenging. An additional complication arises from accurately estimating R, requiring information on the number of donors transferring energy, as well as the number of acceptors distributed across progressively longer distances.

As energy transfer is expected to occur in a time window smaller than that of 2DLP single exciton decay (i.e., $\tau_{\mathrm{ET}}<\tau_{X,2\mathrm{DLP}}$), we can overcome this issue and estimate an energy transfer lifetime by normalizing 2DLP microcrystal decays in the absence and presence of the acceptor. Normalization is performed at long delay times, where recombination is expected to be predominantly single excitonic [56]. This approach results in energy transfer lifetimes of $\tau_{\mathrm{ET},C8}\approx 640\pm 90$ ps for $C_{8}$ heterostructures (see Figure S12), which increases to $\tau_{\mathrm{ET},C18}\approx 1040\pm 170$ ps for $C_{18}$ heterostructures (see Figure S13). Therefore, tuning the nanocrystal capping ligands is a tool to control energy transfer timescales. In the following sections, we focus our characterization on $C_{8}$ heterostructures, as they exhibit more pronounced photophysical changes with respect to their pristine counterparts, compared to $C_{18}$ heterostructures.

### 2.4 Energy Transfer at High Fluence

A common strategy to induce nonlinear phenomena in nanomaterials is to increase the excitation fluence. This promotes the formation of multi‐exciton states, in particular biexcitons, which in confined structures are favored by the strong exciton binding energy enhancing Coulomb interactions. In addition to direct laser photoexcitation, energy transfer can affect the average number of excitons per recombination site ⟨N⟩ and influence biexciton formation and recombination. Motivated by the possibility to control nonlinear phenomena with energy transfer, we investigated the efficiency of biexciton recombination in heterostructures. The steady‐state PL spectra of pristine 2DLP and superlattices acquired at high excitation fluence (3300 nJ cm^−2^) do not exhibit any additional emission peak (see Figure S14), which suggests that biexciton recombinations are dominated by non‐radiative pathways such as Auger recombination. In agreement with previous reports, the biexciton lifetime was therefore extracted from time‐resolved measurements by performing tail normalization and subtraction between low and high fluence photoluminescence decays of the pristine material (see Figures S15 and S16) [50, 56, 57, 58]. Following this procedure, we measured biexciton lifetimes $\tau_{\mathrm{XX},2\mathrm{DLP}}\approx 430\pm 20$ ps and $\tau_{\mathrm{XX},\mathrm{SL}}\approx 1040\pm 70$ ps for pristine 2DLP and $C_{8}$‐superlattices, respectively, in good agreement with previous reports [58, 59, 60]. The energy transfer time that we extracted for the heterostructures is $\tau_{\mathrm{ET},C8}\approx 640\pm 90$ ps, which is roughly on a similar timescale as the biexciton recombination from the 2DLP domain. As a consequence, energy transfer can partially depopulate the 2DLP biexciton state and effectively decrease biexciton recombination. Indeed, at high fluence, the measured time‐resolved emission decay of the donor is overall extended compared to the pristine case (bottom, Figure 3b), suggesting a reduced contribution from biexciton recombination.

For the superlattice domain of the heterostructure, the situation is reversed, as energy transfer occurs on a shorter timescale than both single and biexcitonic decays. As a consequence, in the acceptor, the average number of excitons per recombination site increases (i.e., 〈*N*〉_HTS‐SL_ > 〈*N*〉_SL_, considering only CsPbBr_3_ nanocrystals and 3300 nJ cm^−2^ as excitation fluence, 〈*N*〉_SL_ = 1.16). Therefore, the probability to generate biexcitons is higher for superlattices within the heterostructures, and indeed the time‐resolved emission decay is shorter compared to pristine superlattices (Figure 3c bottom). To estimate the excitation fluence at which energy transfer starts to affect biexcitonic recombination more strongly than single exciton recombination, in Figure 3d,e we report effective lifetimes extracted from biexponential fits of time‐resolved emission decays measured as a function of pump fluence (see Figure S17). The effective lifetimes of the donor (measured at 3 eV, Figure 3d) and the acceptor (measured at 2.4 eV, Figure 3e) are compared with their pristine counterparts (see Figure S18 for statistical quantification). A crossover is visible for the donor at 1430 ± 430 nJ cm^−2^ and for the acceptor at 1900 ± 560 nJ cm^−2^ (pink regions in Figure 3d,e). For the donor, after the crossover the energy transfer becomes efficient enough to decrease the effective 〈*N*〉_HTS‐2DLP_, and hence the probability to generate biexcitons (see Figure 3f). As for the acceptor instead, the effective 〈*N*〉_HTS‐SL_ increases and the biexciton contribution to the overall decay rises.

This coupling of the decay channels is very interesting, as it suggests that the 2DLP microcrystal acts as an energy funnel that shuffles the laser excitation into the superlattice domain. Such behavior could be exploited for the development of highly efficient light‐harvesting nanomaterial systems with unique properties, for example controlled downconversion and FRET‐assisted lasing [61, 62, 63]. However, under these experimental conditions, the photophysics of the heterostructures is strongly influenced by non‐radiative Auger recombination processes.

### 2.5 Low‐Temperature Radiative Recombination Enhancement

Two strategies may be followed to avoid the dissipation of excitation through non‐radiative pathways and exploit energy transfer to induce radiative recombinations at any fluence in the acceptor. First, the acceptor lifetime can be tuned to be faster than the energy transfer. Alternatively, Auger recombination can be suppressed.

A cooperative method would be to cool the system to cryogenic temperature, which would shorten the lifetime of the acceptor due to the bright ground excitonic state of weakly confined ${\mathrm{CsPbBr}}_{3}$ nanocrystals [64, 65], as well as suppress phonon populations responsible for Auger efficiency [66, 67].

Figure 4a shows microscope images of a pristine 2DLP microcrystal under 343 nm laser excitation at 293 (left) and 80 K (right). The 2DLP microcrystal appearance changes from blue to warm white with decreasing temperature. The color change originates from the appearance of a broad emission peak centered at 2.4 eV that we assign to self‐trapped excitons (STE in Figure 4b) [68, 69, 70] that goes along with an intensity decrease of the high energy peak originating from free exciton recombinations (FE in Figure 4b, 3.03 eV at 80 K) [71, 72].

In the $C_{8}$ heterostructure case (Figure 4c), the drop of the 2DLP FE peak intensity is even more pronounced (seven‐fold decrease of the integrated peak area versus 2.5‐fold for pristine 2DLP microcrystals, inset in Figure 4d). The quenching is rationalized by decreased donor–acceptor separation upon cooling enhancing energy transfer, consistent with the contraction of the interparticle spacing reported for superlattices at 80 K (e.g., a decrease of ≈5 Å for $C_{8}$‐capped superlattices) [37].

In the temperature range from 293 to 80 K, the measured energy transfer time decreases from $\tau_{\mathrm{ET}}=640\;\pm \;90$ ps to $\tau_{\mathrm{ET}}=490\;\pm \;30$ ps (Figure 4e, see also Figures S12 and S19), in agreement with our interpretation above. Also, the lifetimes of pristine superlattices shorten compared to room temperature. Specifically, the exciton and biexciton lifetimes at 80 K are $\tau_{X,\mathrm{SL},80K}=500\pm 100$ ps and $\tau_{\mathrm{XX},\mathrm{SL},80K}=300\pm 50$ ps (see Figure 4f,g and Table 2) [58].

**TABLE 2:** Exciton, biexciton, and energy transfer lifetimes at 300 and 80 K.

	293 K		80 K	
	$\tau_{X}$ (ps)	$\tau_{\mathrm{XX}}$ (ps)	$\tau_{X}$ (ps)	$\tau_{\mathrm{XX}}$ (ps)
2DLP	1870 ± 200	430 ± 20	1350 ± 170	390 ± 20
HTS–2DLP	830 ± 170	—	850 ± 100	—
SL	2940 ± 410	1040 ± 70	500 ± 100	300 ± 50
HTS–SL	3520 ± 570	—	690 ± 40	—
Energy transfer	$\tau_{\mathrm{ET}\;-\;C_{8}}=640\pm 90$ ps		$\tau_{\mathrm{ET}\;-\;C_{8}}=490\pm 30$ ps	

Since at 80 K energy transfer is slower compared to the biexciton recombination in superlattices (i.e., $\tau_{\mathrm{XX},\mathrm{SL},80K}<\tau_{\mathrm{ET},80K}$), and comparable to the single exciton lifetime (i.e., $\tau_{X,\mathrm{SL},80K}\approx \tau_{\mathrm{ET},80K}$), it cannot contribute significantly to populate the multi‐exciton states, that is, to increase ${\left\langle N\right\rangle }_{\mathrm{HTS}-\mathrm{SL}}$. In agreement with these findings, Figure 4h shows that even at high fluence the superlattice emission lifetime is longer in the heterostructure as compared to the individual superlattice, which indicates energy transfer to single exciton states with long radiative lifetime (see Figure 4i).

Also, at low temperature, no distinct biexciton emission peak is observed up to 3300 nJ cm^−2^, indicating efficient Auger recombination. However, at low temperature, higher fluence regimes can be explored without compromising the integrity of the heterostructures. At 1.6 mJ cm^−2^, pristine superlattices display an additional red‐shifted emission peak (≈ 30 meV) with superlinear fluence dependence (Figure S20). This peak, attributed to biexciton emission, is also observable from heterostructures at approximately the same fluence, and it is characterized by the similar decay dynamics (Figure S21). The similar behavior of the biexciton emission in isolated superlattices and heterostructures is consistent with the timescales reported above: the superlattice biexciton state is not influenced by energy transfer as it occurs on faster time scales. To achieve energy funneling into the biexciton also at low temperatures the energy transfer dynamics need to be accelerated, which could be achieved by engineering the architecture and/or composition of the heterostructures (i.e., toward more efficient FRET processes).

## 3 Conclusion

In this work, we demonstrated the heterogeneous nucleation of CsPbBr_3_ nanocrystal superlattices triggered by 2DLP microcrystals. This simple approach leads to 2DLP–superlattice heterostructures of various morphologies. The geometric configuration and spectral overlap provide unique opportunities to exploit energy transfer occurring from the 2DLP domain to the superlattices. The large 2DLP domain collects the light excitation and acts as a funnel that directs the energy to the superlattice domain, enhancing either the biexcitonic or single‐excitonic recombination, which can be controlled via the excitation fluence and the sample temperature. These heterostructures represent exciting platforms that could lead to the development of efficient light‐harvesting systems inspired by natural complexes, in which the photon absorption is performed by arrays of chromophores that redirect the energy to photo‐reactive centers with nanoscale precision. In addition, it paves the way toward the deterministic design of heterogeneously grown nanocrystal superlattices, as the versatility of the methods suggests that it could be expanded to a large library of 2D materials and nanocrystals. For example, extending the approach to perovskite nanocrystals and 2D materials with different compositions and thicknesses could improve the spectral overlap, thereby enhancing energy and charge transfer processes. In addition, the optical coupling dynamics observed here may provide a platform to investigate halide migration phenomena or stabilization effects triggered by heterostructure formation. Incorporating plasmonic materials, on the other hand, could enable electromagnetic field enhancement and thereby alter (amplify or quench) the optical response of the semiconducting domain. In a broader context, our heterogeneous nucleation approach could be employed to promote the self‐assembly of nanoparticles. For example controlling the conformation of organic molecules at the surface of the seed crystal, such as bifunctional organic compounds, could enable the creation of anchoring sites suitable for the assembly of nanoparticles or organic semiconductors.

## 4 Experimental Section

### 4.1 Chemical and Reagents

Lead (II) bromide (PbBr_2_, ≥ 98%), cesium carbonate (Cs_2_CO_3_, 99%), hydrobromic acid (HBr, 48%), phenethylamine (PEA, 99%), oleylamine (OLAm, $C_{18}$, 70%), octylamine ($C_{8}$, 99%), oleic acid (OA, 90%), acetonitrile (anhydrous, 99.8%), ethyl acetate (anhydrous, 99.5%), 1‐octadecene (ODE, 90%), and toluene (anhydrous, 99.7%) were purchased from Sigma‐Aldrich and used without further purification.

### 4.2 Synthesis of PEA_2_PbBr_4_ Microcrystalline Powders

To obtain 2DLP PEA_2_PbBr_4_ microcrystalline powders, we followed previously developed protocols with minor modifications [4, 35]. The first step was carried out in a 7 mL glass vial by dissolving 92 mg of PbBr_2_ (0.25 mmol) in 200 μL of HBr, which was then followed by dilution with 2 mL of acetone. Then, 75 μL (0.6 mmol) of PEA (phenethylamine, 99%) was added to the solution to trigger the nucleation of PEA_2_PbBr_4_ microcrystals. The mixture was stirred for ≈3 h to ensure a complete reaction, and the microcrystals were then recovered by centrifuging the vial at 6000 rpm for 3 min, and the supernatant was discarded. The precipitate was redispersed in 2 mL of acetone, and centrifuged again under the same conditions. The washing procedure was repeated two more times, and the resulting solid powder was dried under vacuum for 1 h. The final PEA_2_PbBr_4_ powder was stored in a nitrogen glovebox.

### 4.3 Crystallization of PEA_2_PbBr_4_ Microcrystals on Solid Substrates

A PEA_2_PbBr_4_ stock solution was prepared by dissolving 10 mg of PEA_2_PbBr_4_ microcrystalline powder in 20 mL of acetonitrile. In a 7 mL glass vial, 2 mL of the stock solution was mixed with 2 mL of toluene, which induced the precipitation of the PEA_2_PbBr_4_ microcrystals [36]. The vial was then placed on a metal block pre‐heated to 100 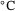 and left for approximately 3 min to ensure complete dissolution of the microcrystals. A 2 cm × 2 cm glass or silicon substrates were placed in glass Petri dish and 170 μL of solution was dropcast on them. The solution was allowed to slowly evaporate for ≈5 h.

### 4.4 Synthesis of CsPbBr_3_ Nanocrystals

The synthesis of $C_{8}$‐ and $C_{18}$‐capped CsPbBr_3_ nanocrystals was performed following previously reported protocols with minor modifications [37, 38]. In a 20 mL glass vial, 72 mg of PbBr_2_ (0.20 mmol) together with 5 mL of ODE, 50 μL (for $C_{18}$) or 150 μL (for $C_{8}$) of OA, and 1.5 mmol of amine (500 μL for $C_{18}$ or 250 μL for $C_{8}$). The vial was placed in a metal block on top of a heating plate, preheated to 185 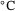. The mixture was heated up to 175 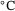 (≈5 min to ensure the dissolution of the PbBr_2_), after which the vial was lifted from the block and fixed with a clamp above it.

The solution was allowed to cool to the desired injection temperature (160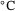 for $C_{18}$ or 170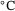 for $C_{8}$), after which 0.5 mL of cesium oleate stock solution was injected. The cesium oleate stock solution was previously prepared in a 40 mL glass vial by dissolving 400 mg of Cs_2_CO_3_ (1.2 mmol) in 15 mL of ODE and 1.75 mL of OA (5.5 mmol) at 120 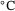, under nitrogen for approximately 1 h. Ten seconds after injection, the reaction was quenched by immersion in an ice‐water bath while stirring. The solution was transferred to a plastic tube and centrifuged for 5 min at 7000 rpm.

For $C_{18}$‐capped nanocrystals, the supernatant was discarded and the precipitate was centrifuged at 5000 rpm for 4 min and the residual liquid was collected with a lint‐free tissue. This cleaning step was repeated once more, after which the precipitate was dissolved in 500 μL of anhydrous toluene. For $C_{8}$‐capped nanocrystals, the supernatant was collected in a plastic tube and 7 mL of anhydrous ethyl acetate was added to it. The solution was centrifuged for 5 min at 7000 rpm and the supernatant discarded. The precipitate was centrifuged two additional times at 5000 rpm for 4 min to remove residual liquid, after which the precipitate was dissolved in 500 μL of toluene. Both $C_{8}$‐ and $C_{18}$‐capped CsPbBr_3_ nanocrystal solutions were centrifuged at 7000 rpm for 5 min to remove any solid aggregate and the supernatant was stored in a glass vial. Before each experiment, the solution was centrifuged to eliminate any solid precipitate.

### 4.5 Preparation of CsPbBr_3_ Nanocrystal Superlattices

Superlattices were prepared on top of a 2 cm × 2 cm glass or silicon substrates that were previously rinsed with acetone, isopropanol, and toluene. Substrates were placed within Petri dishes with a small tilt to ensure both an evaporation rate gradient and a concentration gradient on the substrate. After dropcasting 130 μL of the nanocrystal solution, the Petri dish was closed to ensure slow solvent evaporation.

### 4.6 Preparation of CsPbBr_3_ Superlattice ‐ PEA_2_PbBr_4_ Microcrystal Heterostructures

For the assembly of heterostructures, three of the same 2 cm × 2 cm glass or silicon substrates, on which PEA_2_PbBr_4_ microcrystals were previously grown, were placed in a large glass Petri dish (diameter of ≈ 8 cm). The specific number of substrates was chosen to ensure an appropriate evaporation time. Substrates were slightly tilted by laying one edge on a 1 mm thick glass slab (tilt angle ≈ 3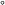). CsPbBr_3_ nanocrystal solutions were diluted with anhydrous toluene. For the $C_{18}$ solution, a 1:1 dilution ratio was performed (i.e., same volume of nanocrystal solution mixed with toluene), while for the $C_{8}$ one, the solution‐to‐toluene dilution ratio was 1:2, as the synthesis typically produced a larger amount of nanocrystals. The typical concentration of the nanocrystal solution was estimated to be approximately 1.9 μM. Then, 130 μL of the CsPbBr_3_ nanocrystal solution was dropcast on top of each film, and the Petri dish was closed. The formation of heterostructures in the upper part of the substrate occurs in ≈ 6 h. The lower part of the substrate is partially wet and would require 1 or 2 additional hours at ambient pressure to dry completely. However, to remove the organic material in excess so to avoid the potential degradation of the heterostructures, the substrates were placed in a desiccator and dried under vacuum overnight. At the end of the evaporation, for the lower and upper parts of the substrate, at λ= 400 nm, we measured optical densities of ≈ 0.6 and 0.3, respectively.

### 4.7 Diffraction Data Collection

The θ:2θ XRD patterns were acquired on a Panalytical Empyrean diffractometer equipped with a 1.8 kW Cu Kα ceramic X‐ray tube and a PIXcel3D 2 × 2 area detector operating at 45 kV and 40 mA.

### 4.8 Morphology Characterization

The morphology of the heterostructures was investigated by means of an optical profilometer (ZETA), of a Zeiss GeminiSEM 560 (Zeiss, Oberkochen, Germany) field‐emission scanning electron microscope (SEM), equipped with a gun operating at 10 kV acceleration voltage, and a Helios G4 UX Dual Beam SEM instrument operating at 10 kV.

### 4.9 Equilibrium Optical Measurements

Micro‐PL measurements were performed using an optical imaging and spectroscopy system coupled with an inverted microscope (Nikon, Eclipse Ti). To excite the samples, a femtosecond laser was employed (NKT, Origami). The laser pulses (1030 nm) were first frequency doubled and subsequently tripled by means of beta barium oxide (BBO) second harmonic generation, and third harmonic generation crystals to obtain a 343 nm excitation wavelength. The excitation laser was focused on the sample using an objective (Nikon, 10×/0.25 numerical aperture). The emission was collected with the same objective and was measured using a fiber‐based spectrometer (Ocean Insight, USB2000)

### 4.10 Time‐Resolved Measurements

Time‐correlated single‐photon counting measurements were conducted using the frequency‐tripled laser focused on the sample by means of a 10× objective. The emission was collected with the same objective and was directed into a Gemini interferometer coupled to a single photon counting avalanche photodiode (Micro Photon Devices). Signals from the avalanche photodiode were analyzed using a time‐correlated single photon counting module (Picoquant, PicoHarp 300).

## Conflicts of Interest

The authors declare no conflicts of interest.

## Supporting information


**Supporting File**: adma74290‐sup‐0001‐SuppMat.pdf.

## Data Availability

The data reported in this manuscript are available from the corresponding author upon reasonable request.
