# Supplementary material for: Deterministic Nucleation of Nanocrystal Superlattices on 2D Perovskites for Light‐Funneling Heterostructures
Source: Adv Mater. 2026 Aug 6;38(52):e74290. doi: 10.1002/adma.74290 (PMC13579231; doi:10.1002/adma.74290)
Supplement: Supplementary file 1 [file ADMA-38-e74290-s001.pdf]

Supporting Information for
Deterministic nucleation of nanocrystal superlattices on 2D perovskites for light-funneling heterostructures

Umberto Filippi,[1,2,*] Alexander Schleusener,[1] Simone Lauciello,[1] Roman Krahne,[1] Dmitry Baranov,[3] Liberato Manna,[1] and Masaru Kuno[2,4]

[1] *Department of Nanochemistry, Istituto Italiano di Tecnologia, 16163 Genova, Italy*
[2] *University of Notre Dame, Department of Physics and Astronomy, Notre Dame, IN 46556 USA*
[3] *Division of Chemical Physics and NanoLund, Department of Chemistry, Lund University, P.O. Box 124, Lund, SE-221 00 Sweden*
[4] *University of Notre Dame, Department of Chemistry and Biochemistry, Notre Dame, IN 46556 USA*

CONTENTS

S1. 2DLP microcrystal lateral size as a function of temperature 2
S2. *In situ* characterization of heterostructure growth 3
S3. Heterostructure morphology 4
S4. Disorder as a function of the heterostructure position on the substrate 7
S5. Room temperature optical properties of C_{18} heterostructures 9
S6. Calculation of energy transfer parameters 9
S7. Fluence Dependent Photoluminescence Spectra 11
S8. Calculation of biexciton lifetimes 11
S9. Time-resolved emission decay fitting 13
S10. Cryogenic optical characterization 14
References 16

* umberto.filippi@iit.it

S1. 2DLP MICROCRYSTAL LATERAL SIZE AS A FUNCTION OF TEMPERATURE

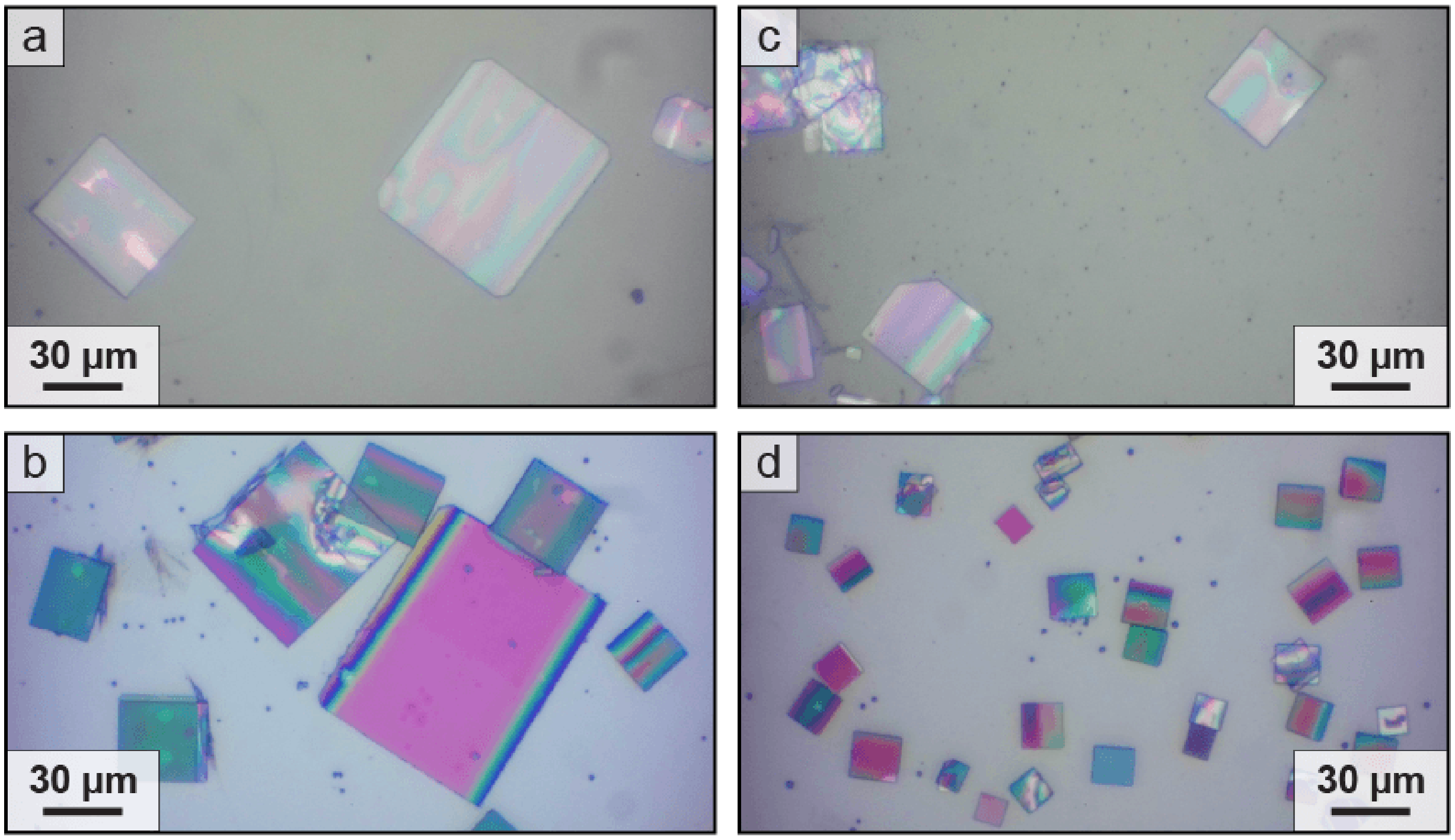

Figure S1: a-d) Microscope images of two-dimensional layered perovskite (2DLP) microcrystals obtained by heating the precursor solution at different temperatures: (a) and (b) at 90 °C, (c) and (d) at 60 °C. As observable, higher temperature favors the nucleation of larger microcrystals.

S2. *IN SITU* CHARACTERIZATION OF HETEROSTRUCTURE GROWTH

As specified in the main text, the heterostructure properties depend on the region of the substrate where the heterostructures form. Before dropcasting the $CsPbBr_3$ nanocrystal solution, the substrate, on which the 2DLP microcrystals were previously grown, is tilted in order to create a nanocrystal concentration gradient. As a result, differently from what we reported in Figure 1d in the main text, in the lower regions of the substrate, it is possible to observe heterostructures that retain the emission peak assigned to the quasi-2D microcrystals formed during the heterostructure growth (see **Figures S2a,b**).
Another difference that can be observed depending on the location on the substrate is the change in the emission peak intensity of $CsPbBr_3$ nanocrystals during the heterostructure formation. The factors affecting this behavior include the number of emitting nanocrystals, the nanocrystal density and the quantum yield.
The latter is expected to decrease (independently of the substrate region) as the nanocrystals transition from being dispersed in solution to the solid state (as they precipitate onto the film).[1] In contrast, the number of emitting nanocrystals and their density increase in the lower region of the substrate, as the solution accumulates due to gravity and gradually evaporates. As a consequence, the intensity of the emission from $CsPbBr_3$ nanocrystals increases with the evaporation time (**Figure S2a**).
In the upper part of the substrate, the nanocrystal density also increases with time as the solvent evaporates. However, the number of emitting nanocrystals can decrease with time as the solution progressively migrates toward the bottom part of the substrate. Therefore, the decrease of quantum yield and of the number of emitting nanocrystals, make it possible to observe an overall decrease in the emission peak intensity of $CsPbBr_3$ nanocrystals (as shown in Figure 1d in the main text).

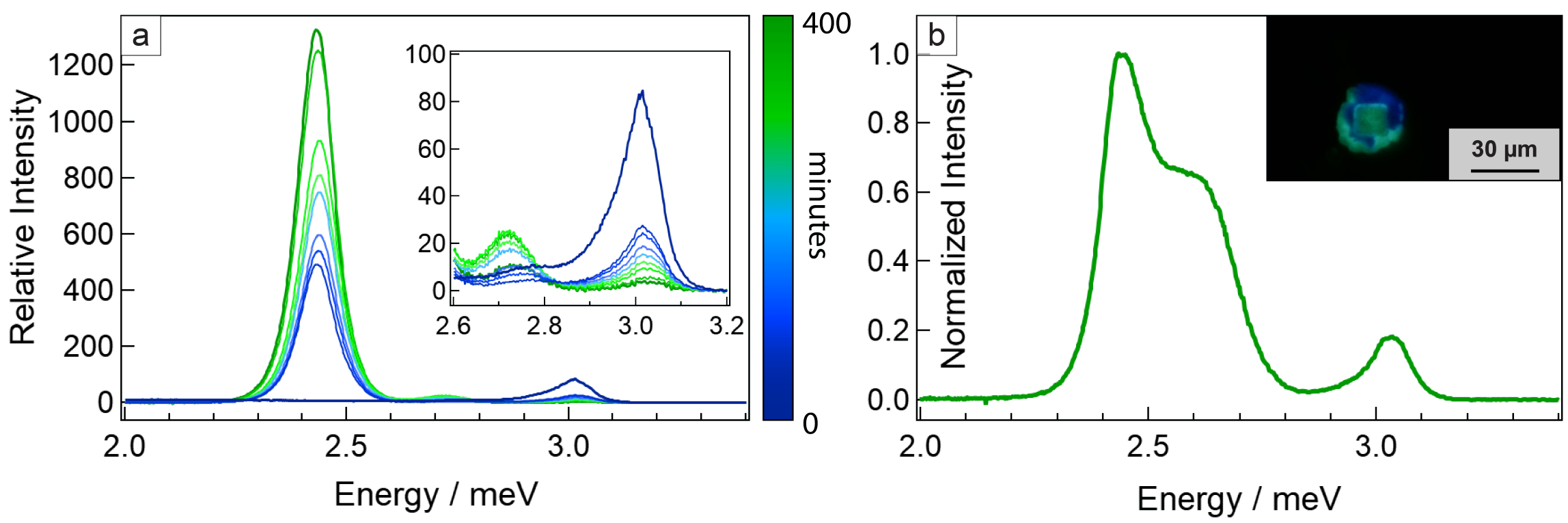

Figure S2: a) Time evolution of the PL spectra during the heterostructure nucleation. Differently from Figure 1c of the main text, the emission at 2.72 eV is still observable after the end of the growth. b) Example of one heterostructure showing the emission peak of a 2DLP microcrystal with n>1.

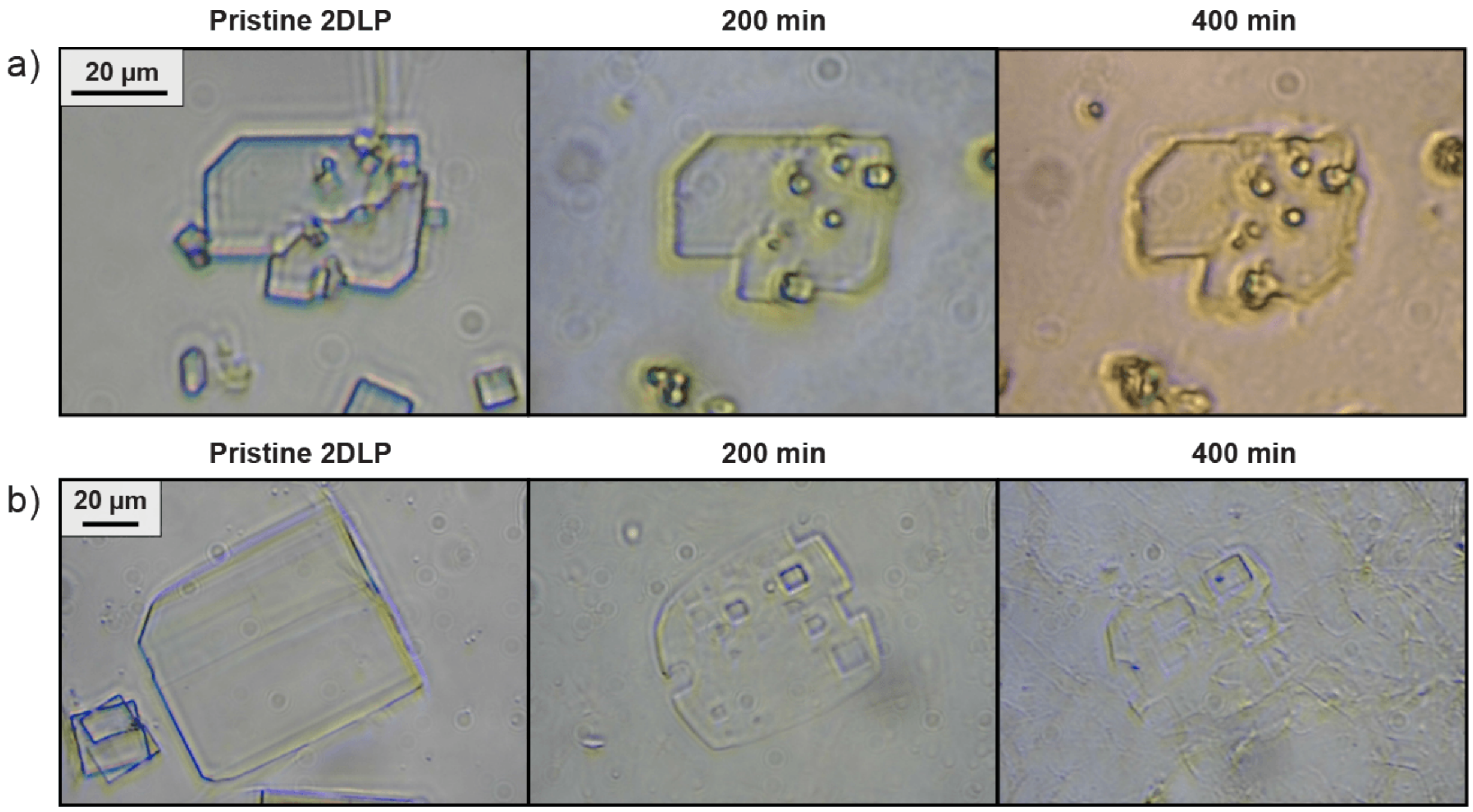

Figure S3: a,b) Time evolution of 2DLP microcrystals after dropcasting the $CsPbBr_3$ nanocrystal solution. In (a). after an initial edge etching, a superlattice domain grows along the edges. In (b), the 2DLP microcrystal is progressively dissolved.

S3. HETEROSTRUCTURE MORPHOLOGY

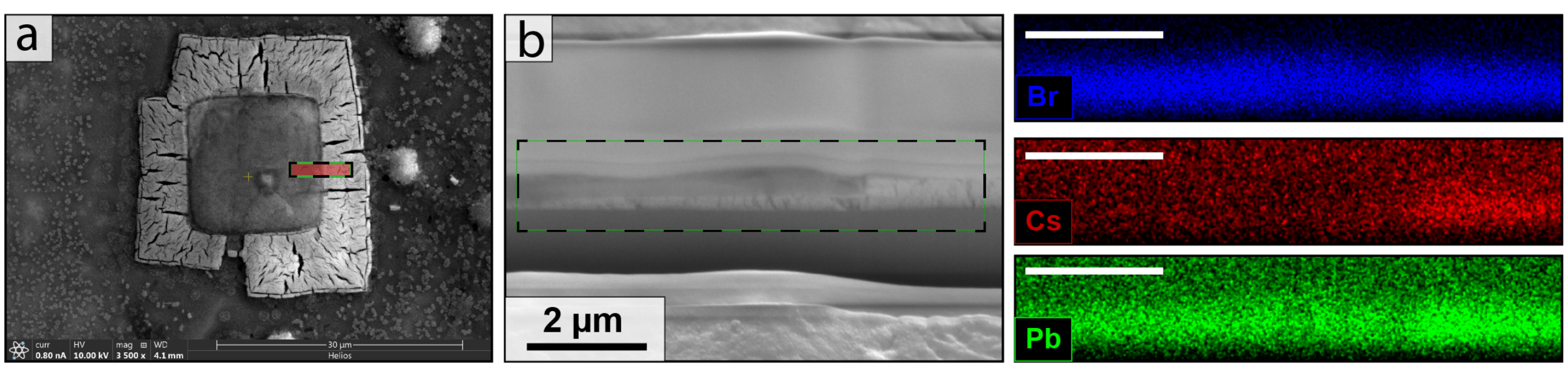

Figure S4: a) SEM image of a core-crown heterostructure. The red box highlights the approximate region removed to obtain the cross-sectional view (box not to scale). b) Cross-sectional image and corresponding EDX maps acquired at the interface of the heterostructures. In the EDX maps the scale bar is 2 µm.

Under high concentration and long evaporation conditions, that can be achieved for example at the bottom or the edges of the heterostructure film, it is possible to observe heterostructures where nanocrystals assemble also on the top face of the 2DLP microcrystals (**Figure S5**, top part). In some extreme cases, the microcrystals fully dissolve, and trigger the formation of large nanocrystal superlattices reaching up to 85 µm (**Figure S5**, bottom part).

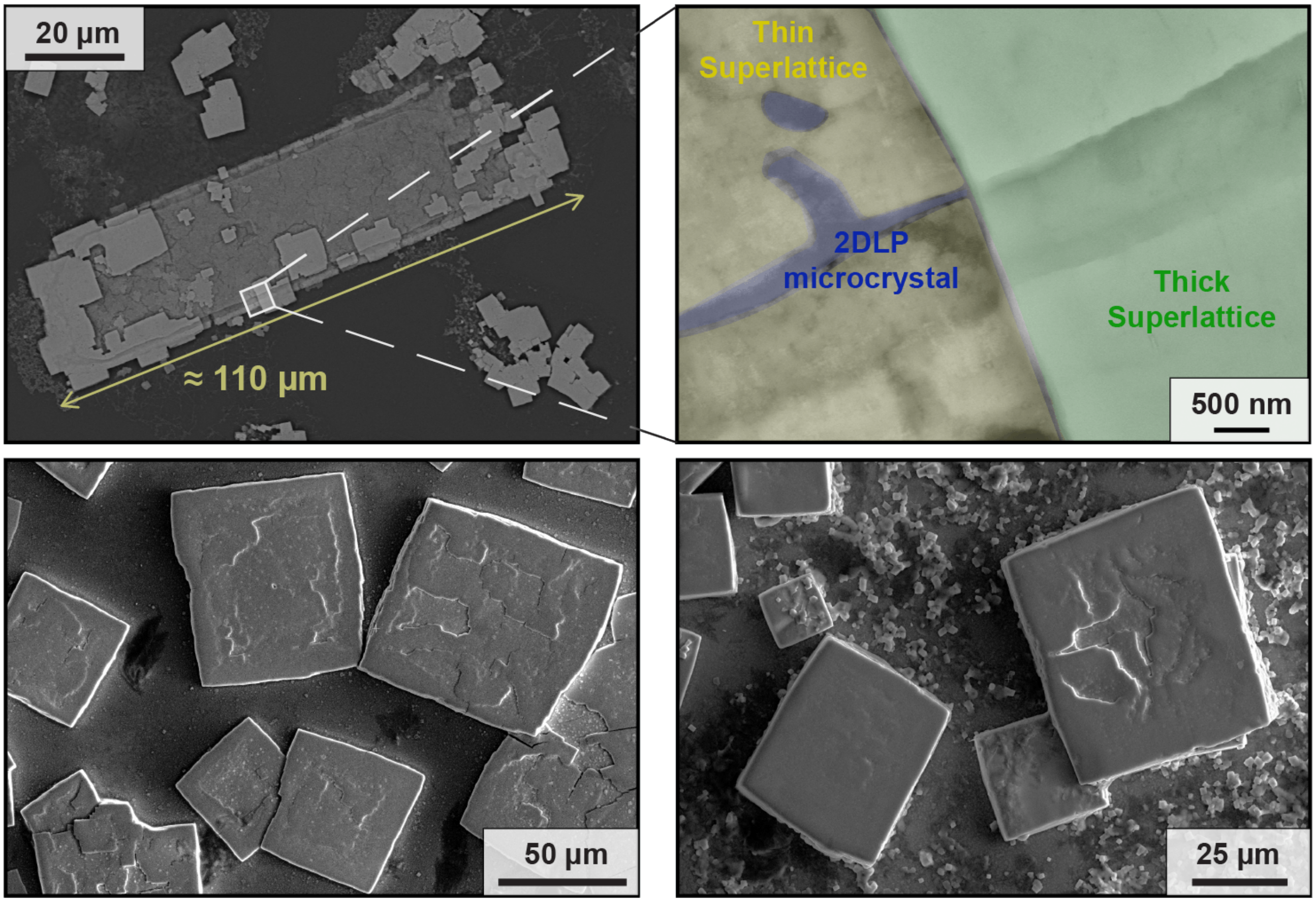

Figure S5: Heterostructure where nanocrystals assembled in correspondence of the lateral and top faces. Large superlattices forming at the edges of the film.

Regarding the quasi-2D perovskite components, if their emission is not visible in the heterostructure photoluminescence spectrum at the end of the growth process (like for the heterostructures shown in Figures 3 and 4 of the main text), we believe they do not form a stable interface either with the nanocrystals nor with the PEA_2PbBr_4 domains. They form as transient intermediates during the solvent evaporation and heterostructure growth, but detach from the main 2DLP microcrystal and mostly segregate at the edges or in the lower part of the substrate due to the solvent flow. However, their presence can still be observed in some heterostructures, specifically those grown in the lower part of the substrate, like we show in **Figure S2**. Regarding the XRD measurements, the diffraction pattern of the heterostructures is dominated by reflections from the nanocrystal superlattices and the PEA_2PbBr_4 microcrystals, which suggests that they are the dominant components. However, contributions from $(PEA)_2Cs_{n-1}Pb_nBr_{3n+1}$ can be observed at low angles in the XRD pattern, as demonstrated in **Figure S6**, where a C_{18} heterostructure sample is compared with the reference diffraction patterns of pristine PEA_2PbBr_4, $PEA_2CsPb_2Br_7$ and $PEA_2Cs_2Pb_3Br_{10}$. These reflections, with different intensity ratios, are always observed in our measurements.

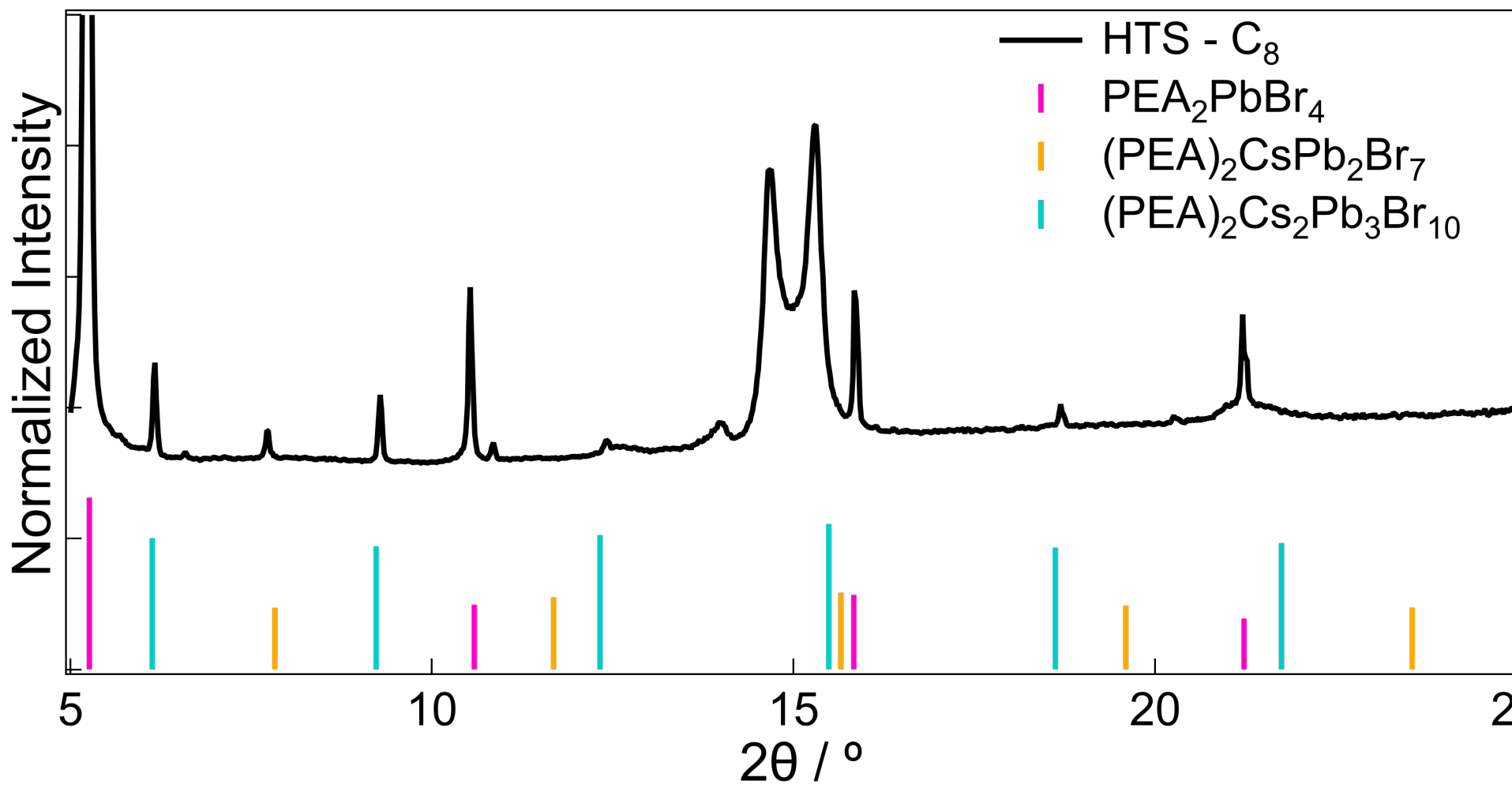

Figure S6: a) XRD pattern of a C_8 heterostructure sample showing residual signatures of $(PEA)_2Cs_{n-1}Pb_nBr_{3n+1}$ mixed-phase domains.

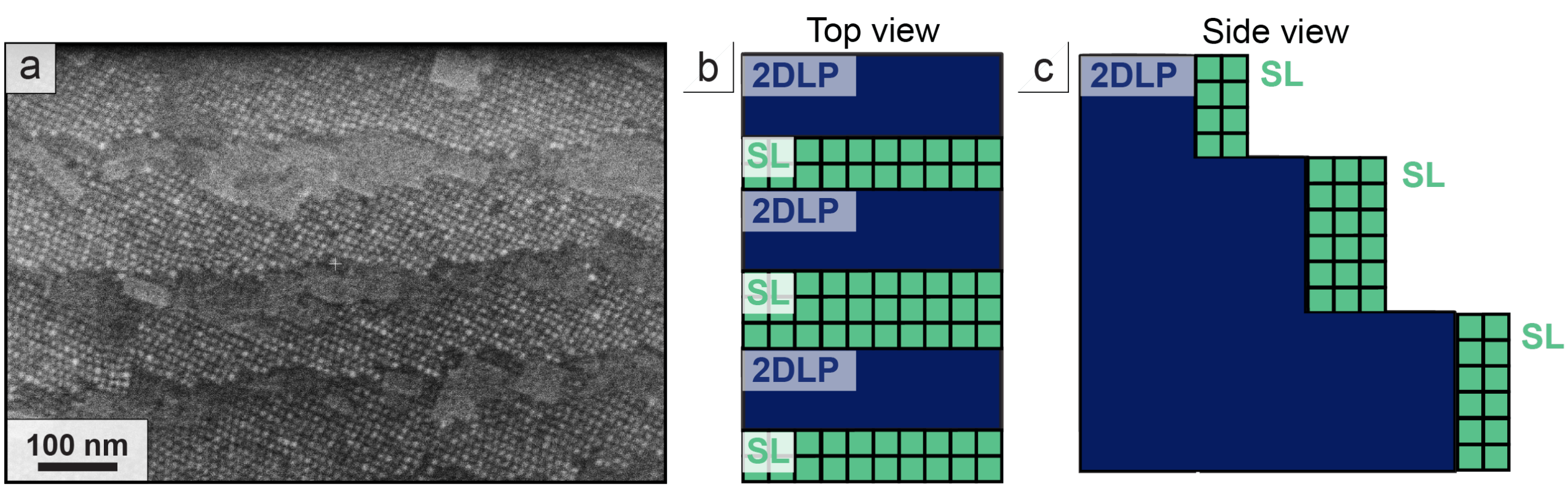

Figure S7: a) Alternating sections of nanocrystals and 2DLP sheets. b,c) Schematic representations of the top (b) and side (c) views.

S4. DISORDER AS A FUNCTION OF THE HETEROSTRUCTURE POSITION ON THE SUBSTRATE

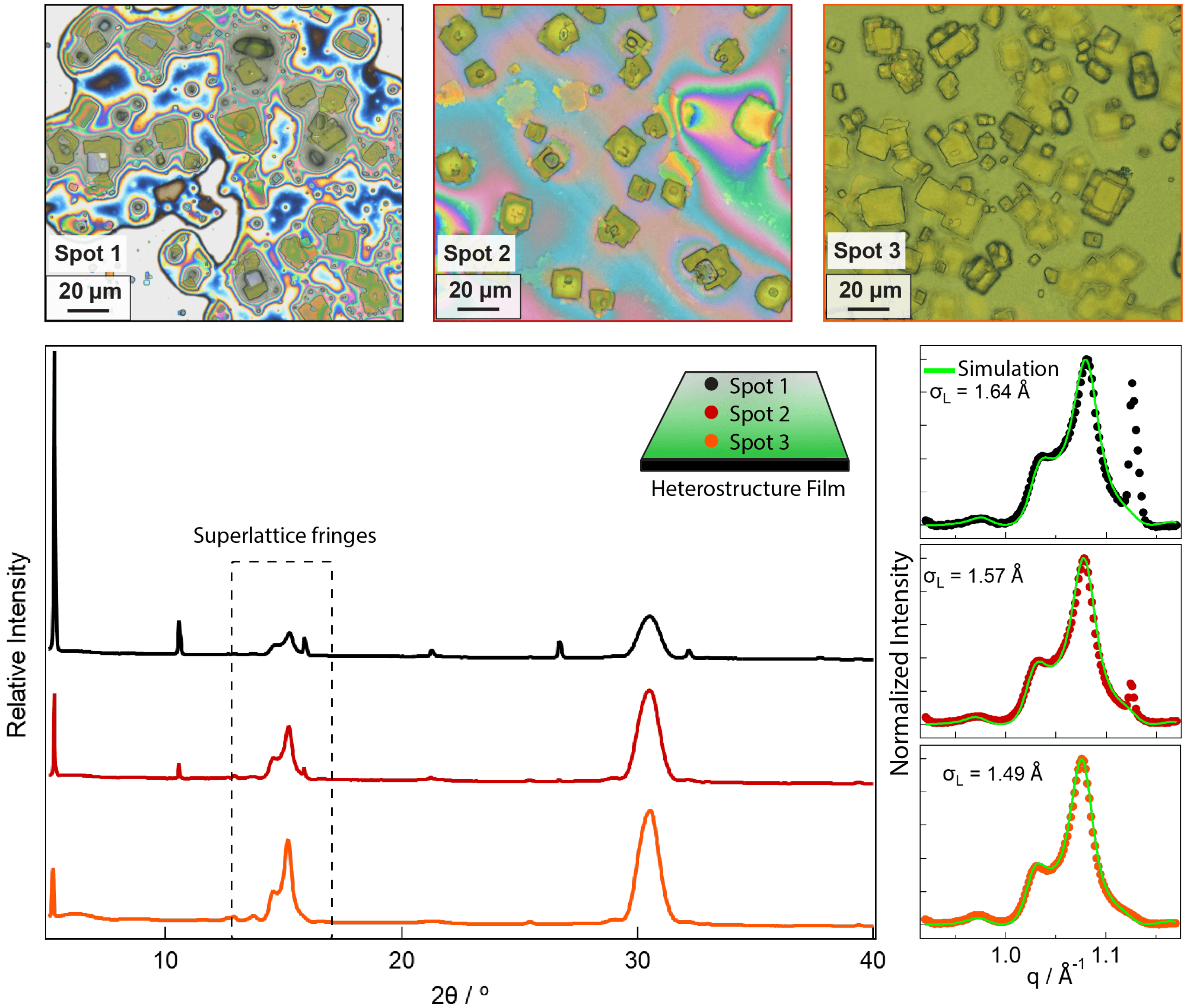

Figure S8: Microscope images and XRD patterns acquired at different positions of the heterostructure film which was prepared by dropcasting the nanocrystal solution on a tilted substrate, to create a concentration gradient. The images show that the top part of the film is characterized by well defined and isolated heterostructures, which progressively become more covered by nanocrystals and eventually the bottom of the film is characterized by a continuous layer of superlattices. The XRD patterns at the same time display broader fringes on the top of the film compared to the bottom, which suggests higher structural disorder.

To investigate the structural effects induced by these recrystallization and exchange processes on the nanocrystals, we performed XRD measurements along different positions of the heterostructure film. As we discussed in the main text, because the nanocrystal solution is dropcast onto a tilted substrate, the upper region is characterized by heterostructures formed from a less concentrated solution of nanocrystals, while the lower region experiences larger concentration due to particle accumulation driven by gravity. This difference results in isolated structures in the former region and a continuous film in the latter one. The XRD signal from the upper domain shows broader superlattice fringes in correspondence of the first Bragg reflection at $q = 1.05$ Å^{-1} (**Figure S8**). When fitted using the multilayer diffraction fitting routine developed in previous works for the superlattice analysis,[2, 3] it yields a larger value of the average misplacement parameter σ_L (1.64 Å vs 1.49 Å). This indicates a greater stacking disorder of the nanocrystals which could arise from the suboptimal superlattice growth condition (as they are force to growth on the 2DLP microcrystals) or from the partial exchange of ligands between 2DLP and nanocrystals (e.g., phenethylamine replacing

oleylamine). The latter possibility could represent an intriguing approach to transfer properties between materials. To test these hypotheses, we assembled heterostructures using octylamine-capped (C_8) $CsPbBr_3$ nanocrystals. This passivation strategy was already employed in our previous work to enhance the structural order of the assemblies, with the drawback of order loss under vacuum, due to the volatility of the ligands.[4] In the heterostructures case the order is not only enhanced but also it is preserved after vacuum, which can be interpreted as an evidence of partial ligand exchange (C_8 with PEA) in the nanocrystals (**Figure S9**).

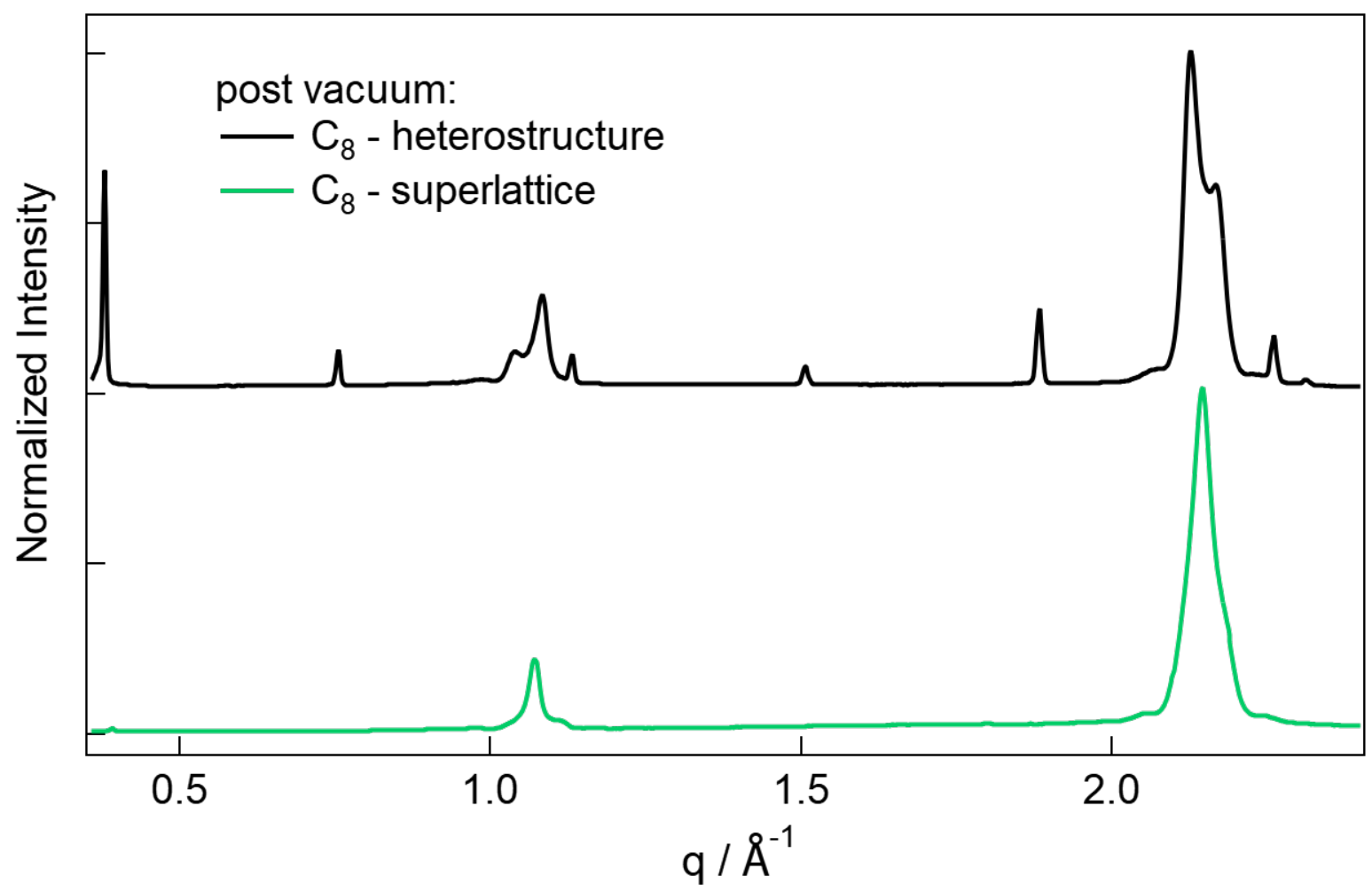

Figure S9: XRD pattern of C_8-capped heterostructures and superlattices before and after vacuum. The superlattice fringes in correspondence of the Bragg peaks are lost for the superlattice sample, as some of the volatile ligands are removed when vacuum is applied. For the heterostructure case instead the fringes are still observable, and this suggests that ligand exchange occurs between the 2DLP microcrystals and the superlattices (octylamine exchanged for phenethylamine).

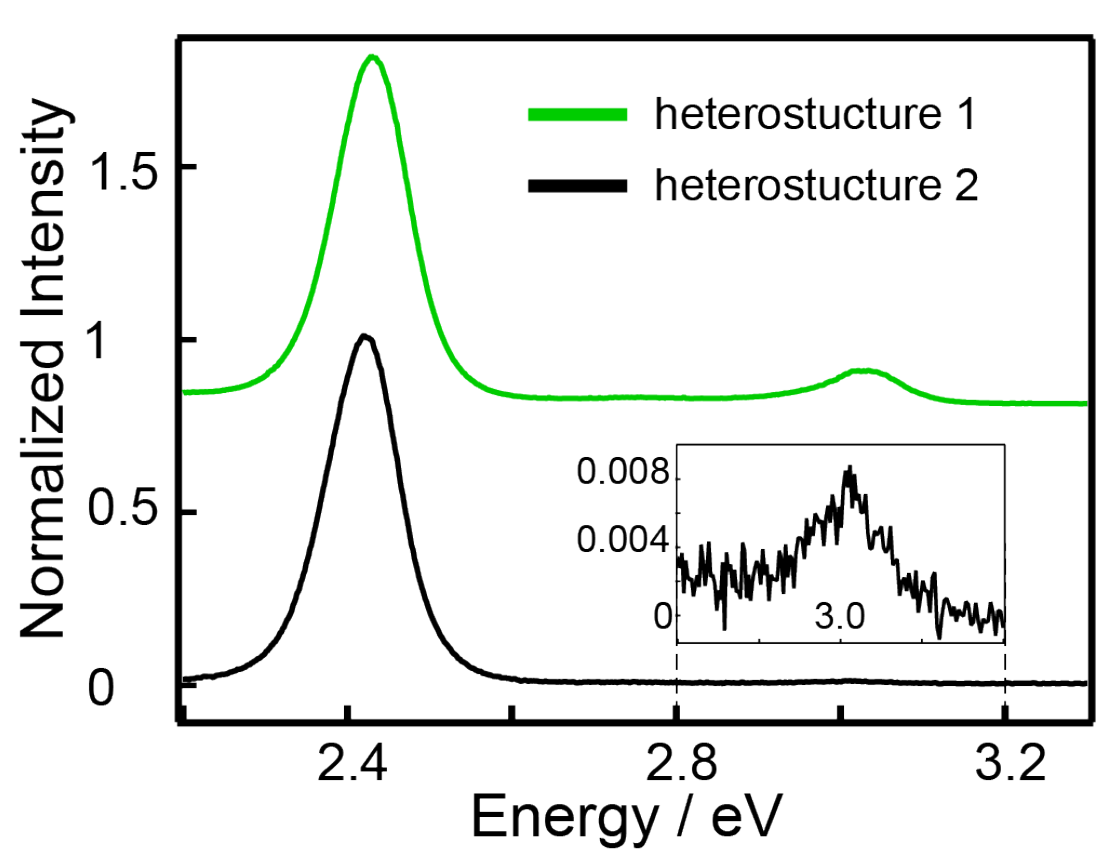

Figure S10: Photoluminescence spectra of two different heterostructures. Top trace shows both the superlattices and 2DLP emission peaks. Bottom spectrum instead exhibit a very weak emission from the 2DLP domain.

S5. ROOM TEMPERATURE OPTICAL PROPERTIES OF C_{18} HETEROSTRUCTURES

Figure S11 shows the time-resolved emission decays of pristine 2DLP PEA_2PbBr_4 microcrystals, pristine C_{18} superlattices and C_{18} heterostructures, extracted at 3 eV and 2.4 eV. At 3 eV, in the high fluence regime, we observed the extension of the donor decay compared to the pristine case, as observed for C_8 heterostructures. At 2.4 eV, in the low fluence regime, the acceptor shows a mild extension of the decay compared to pristine C_{18} superlattice. In the high fluence regime, the comparison is reversed, and the acceptor becomes faster than the pristine C_{18} superlattice. This is compatible with the enhanced probability of generating biexcitons in the acceptor compared to the pristine superlattice, as reported for C_8 heterostructures.

With respect to the C_8 case, for C_{18} we observed the shortening of the acceptor decay with respect to the pristine superlattice at lower fluences (1290 nJ/cm^2 instead of 3300 nJ/cm^2). This is expected, as C_{18}-capped nanocrystals have smaller edge length compared to C_8-capped nanocrystals (8 nm compared to 9.5 nm), and this enhances Auger recombination, which is expected to scale with the cube of the nanocrystal radius.

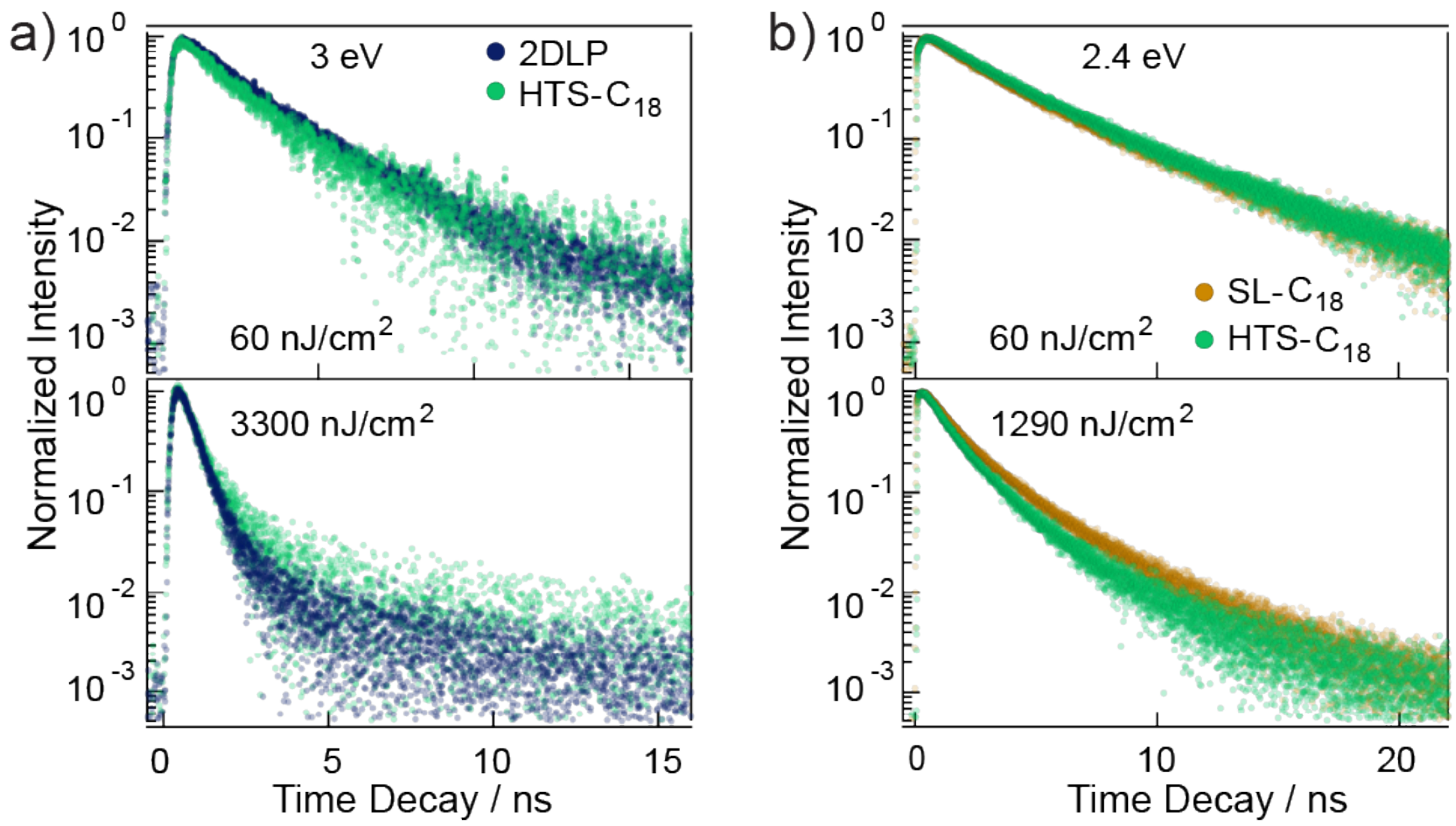

Figure S11: a) Time-resolved emission decays of a pristine 2DLP microcrystal and a C_{18} heterostructure, extracted at 3 eV, collected in the low-fluence (60 nJ/cm^2, top) and high-fluence (3300 nJ/cm^2, bottom) regimes. b) Similar comparison for traces extracted at the $CsPbBr_3$ nanocrystal emission peak ($\approx$ 2.4 eV) for a pristine C_{18} superlattice (orange line) and a C_{18} heterostructure (spring green line). Low fluence (60 nJ/cm^2) is shown in the top graph, and high fluence (1290 nJ/cm^2) in the bottom graph.

S6. CALCULATION OF ENERGY TRANSFER PARAMETERS

The Förster radius R_0 was calculated using the equation:

$$R_0 = 0.211 \times \left(\frac{k^2 \eta_{PL}}{n^4} \int_0^\infty F_D(\lambda)\epsilon_A(\lambda)\lambda^4 d\lambda \right), \tag{S1}$$

where k is the orientation factor, which is assumed to be 2/3 for randomly oriented dipoles, $\eta_{PL} = 0.15$ is the PL quantum yield of the donor in the absence of the acceptor, n is the refractive index of the medium. To estimate n, we employed 1.9 for $CsPbBr_3$ (2.2 for the inorganic core and 1.4 for the organic shell, accounting respectively for the 2/3 and of the 1/3 of final n of $CsPbBr_3$),[5, 6] and 2.8 for PEA_2PbBr_4,[7] and estimated $n = 1.9 \times 0.5 + 2.8 \times 0.5 = 2.35$. $F_D(\lambda)$ is the PL spectrum of the pristine PEA_2PbBr_4 2DLP microcrystal with the integrated area normalized to 1 and $\epsilon_A(\lambda)$ is the absorption of the acceptor expressed in $M^{-1}cm^{-1}$.
The integral represents the spectrum overlap integral denoted as $J(\lambda)$ and it results to be $J(\lambda)$ =2.457×10^{17}

$M^{-1}\,nm^4\,mol^{-1}$. The value is particularly large compared with values calculated for example for $CsPbBr_3$ nanocrystals and organic dyes or ligands (5-6 nm)[5, 8, 9]. This can be explained by the complete spectral overlap of the PEA_2PbBr_4 2DLP microcrystal emission with the $CsPbBr_3$ absorption. The resulting value for the Förster radius is $R_0 \approx$67 nm.

As discussed in the main text, a large R_0 suggests that energy transfer occurs from the 2DLP microcrystal to many nanocrystals vertical arrays. Therefore, energy transfer will include different zones, and its nature will change depending on which zone we are considering. The zones are defined with respect to a distance $b \sim \frac{\lambda}{2\pi n}$, where λ represents the donor emission wavelength.[10] The non-radiative energy transfer should occur in the near-field zone between $0.01b$ and $0.1b$ ($\approx$ 2.7 nm) and scale with m =6. The intermediate-zone should extend until $10b$ ($\approx$ 270 nm), scale with m =4 and include both non-radiative and radiative contributions. The far-zone is the area extending beyond $10b \approx$ 270 nm, and it is dominated by radiative energy transfer.

We can calculate the energy transfer rate k_{ET} as,

$$k_{ET} = \frac{1}{\tau_{ET}}, \tag{S2}$$

where τ_{ET} is the energy transfer lifetime, which we estimated by normalizing the time-resolved emission of the pristine 2DLP microcrystal at long decay times, where recombination is expected to be predominantly single excitonic, and eventually subtracting from it the 2DLP microcrystal decay when assembled in the heterostructures (previously normalized with the same approach).

Representative normalized traces are shown in **Figures S12 and Figures S13**. Analysis of three independent measurements yields energy transfer lifetimes of $\tau_{\mathrm{ET}} \approx 640 \pm 90$ ps and 1040 ± 170 ps for the C_8 and C_{18} heterostructures, respectively. We can employ these values to estimate the energy transfer efficiency.

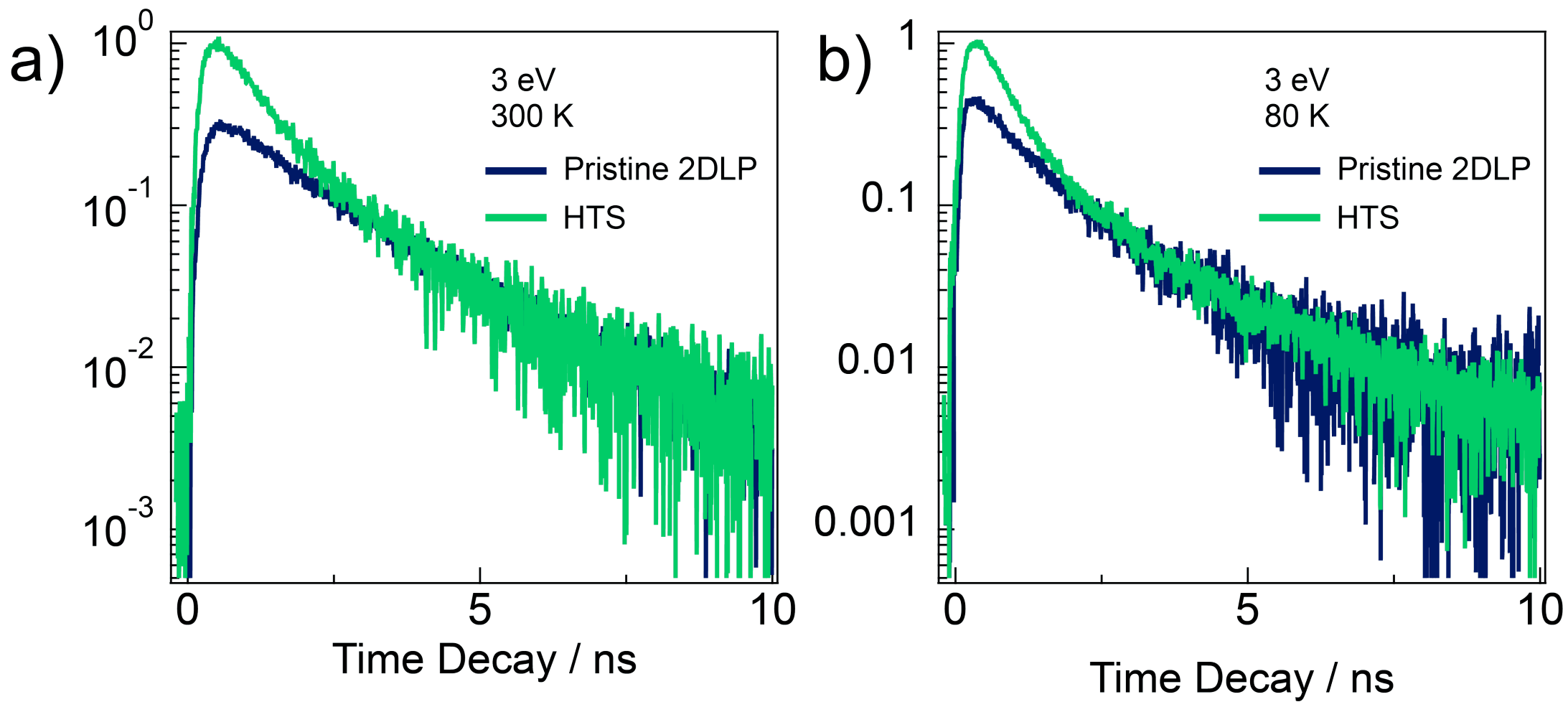

Figure S12: a,b) Representative time-resolved photoluminescence decays measured at 300 K and 80 K respectively for one pristine 2DLP microcrystal (dark blue trace) and one C_8 heterostructure (green trace). The decays are extracted at 3 eV and they are used to calculate the energy transfer decays reported in the main text in Figure 4.

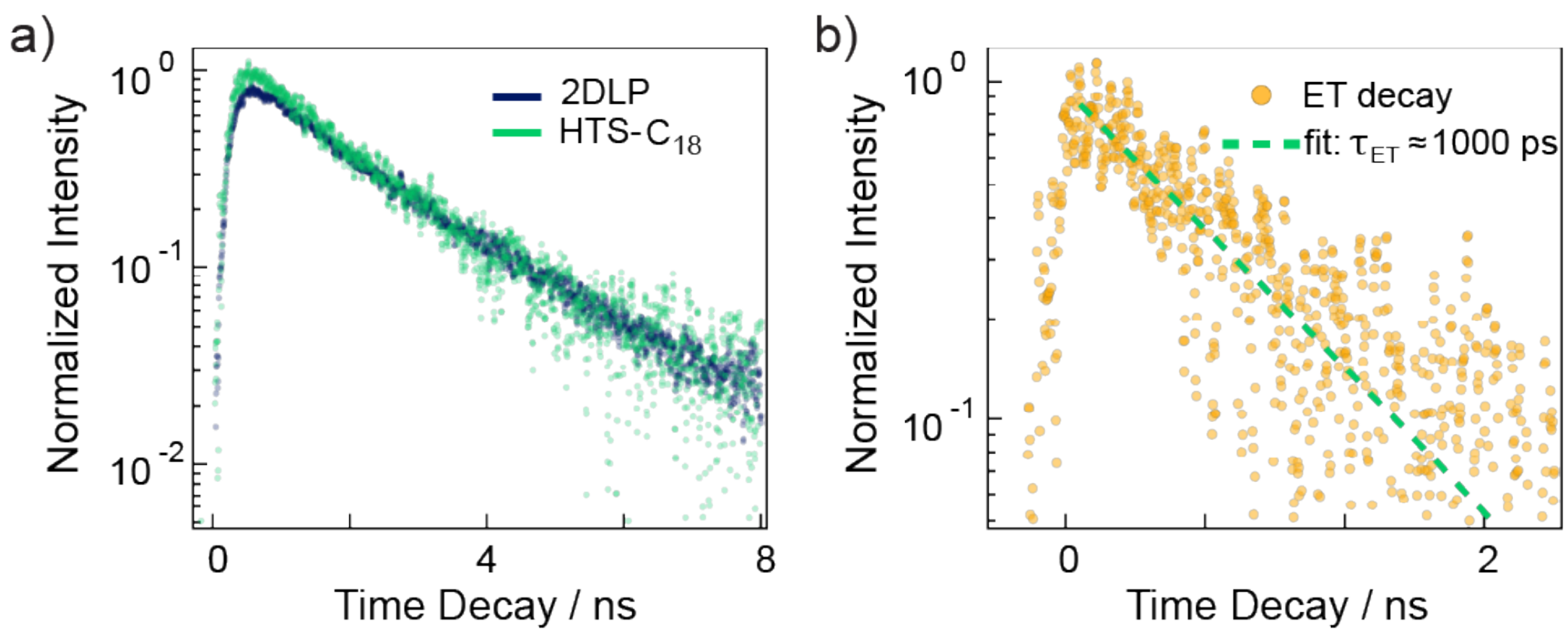

Figure S13: a,b) Tail-normalized time-resolved photoluminescence decays measured for a pristine 2DLP microcrystal and a C_{18} heterostructure (a) and their difference (b), the latter used to calculate the energy transfer decay from 2DLP to C_{18} superlattices.

S7. FLUENCE DEPENDENT PHOTOLUMINESCENCE SPECTRA

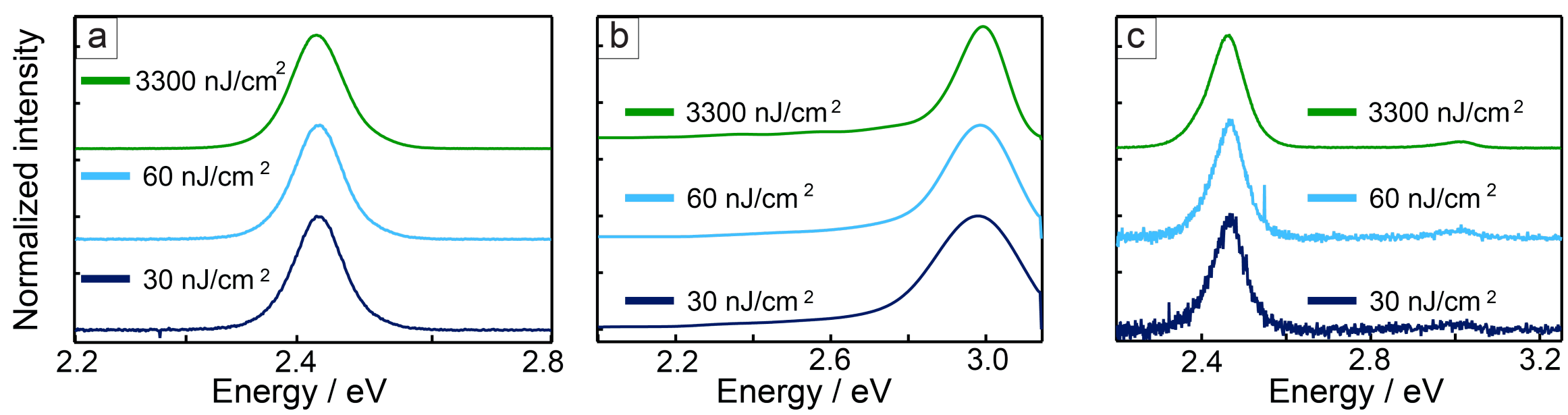

Figure S14: Fluence dependent photoluminescence spectra of (a) one pristine superlattice, (b) one pristine 2DLP microcrystal and (c) a heterostructure.

S8. CALCULATION OF BIEXCITON LIFETIMES

The 2DLP microcrystal biexciton lifetimes $\tau_{2DLP_{XX}}$ were calculated by normalizing the 2DLP microcrystal time-resolved emission measured in the low and high fluence regimes (60 nJ/cm^2 and 3300 nJ/cm^2 respectively) at long decay times, where again the emission is supposed to be single excitonic. Following this procedure, we calculated $\tau_{XX,2DLP}$ = 430 ± 20 ps and 390 ± 20 ps at 293 K and 80 K respectively (see **Figure S15** for a representative calculation of the biexciton lifetime). As observed at room temperature, at 80 K in the high fluence regime (3300 nJ/cm^2) the biexciton contribution is mitigated in the heterostructure case, as a part of the excitation is channeled to the superlattice domain (see Figure S19).

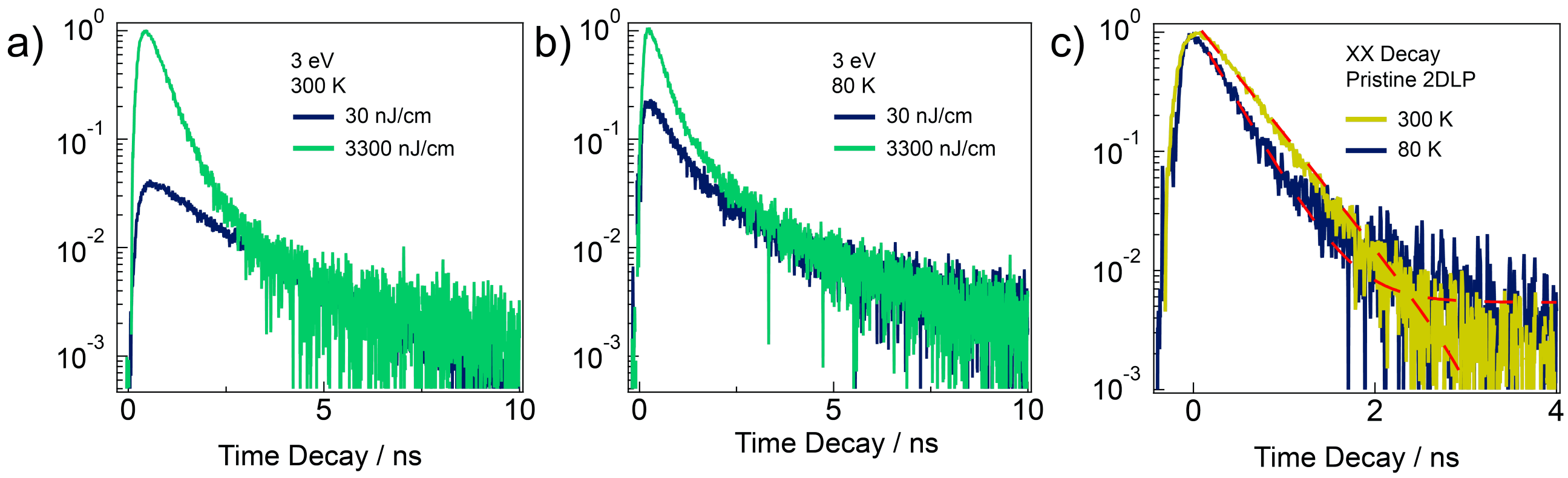

Figure S15: a,b) Time resolved photoluminescence decays measured for pristine 2DLP microcrystals in the low and high fluence regimes at 300 K and 80 K respectively. c) Biexciton decays that were extracted by calculating the difference between the low and high fluence traces in a and b.

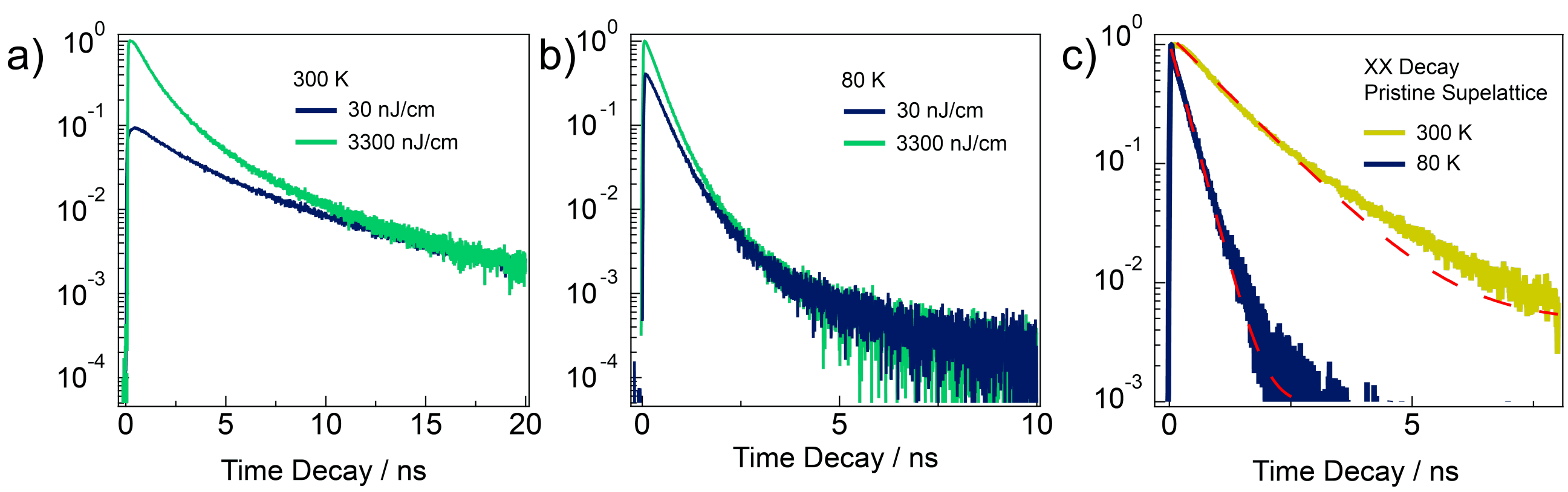

Figure S16: a,b) Time resolved photoluminescence decays measured for pristine superlattices in the low and high fluence regimes at 300 K and 80 K respectively. c) Biexciton decays that were extracted by calculating the difference between the low and high fluence traces reported in a and b.

S9. TIME-RESOLVED EMISSION DECAY FITTING

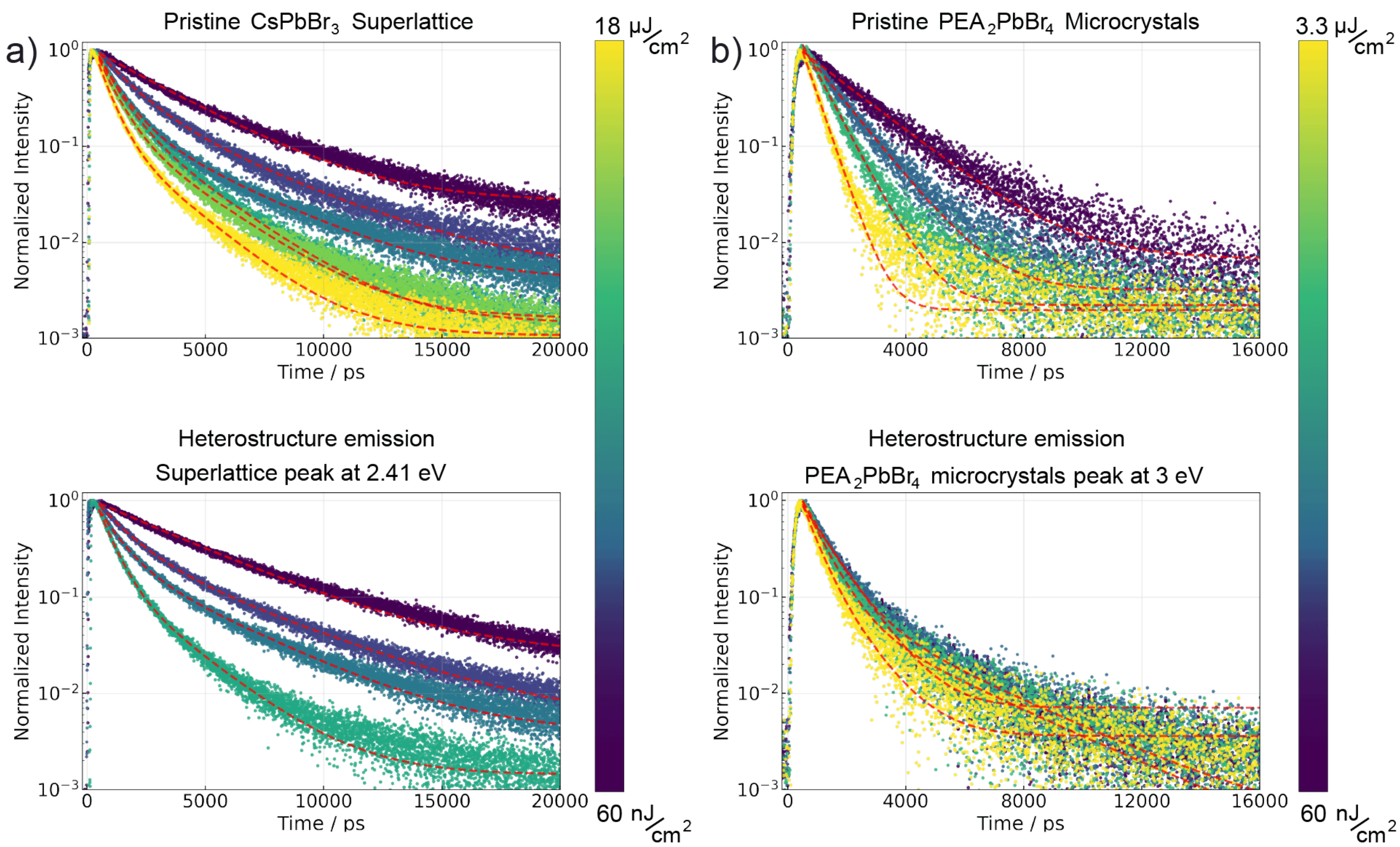

Figure S17: a,b) Time resolved photoluminescence decays measured in correspondence of the $CsPbBr_3$ nanocrystal superlattice emission (a) and of the PEA_2PbBr_4 2DLP microcrystal emission (b) in the case of pristine crystals (top) and of heterostructures (bottom). The decay traces measured at the lowest fluence (60 nJ cm^{-2}, darkest violet traces) are fitted using a single exponential decay function, all the other traces are fitted by mean of equation S3.

The high fluence time-resolved emission decays were fitted using a bi-exponential function expressed as:

$$I(t) = A_1 \exp \frac{-t}{\tau_1} + A_2 \exp \frac{-t}{\tau_2} + c, \tag{S3}$$

where A_n is the amplitudes of the exponential decays and τ_n are the corresponding decay time constants. The fits are shown in Figure S17. The the effective time decay τ_{eff}, reported in Figure 3 of the main text, were calculated using the following expression:

$$\tau_{eff} = \tau_1 \times (\frac{A_1}{A_1 + A_2}) + \tau_2 \times (\frac{A_2}{A_1 + A_2}). \tag{S4}$$

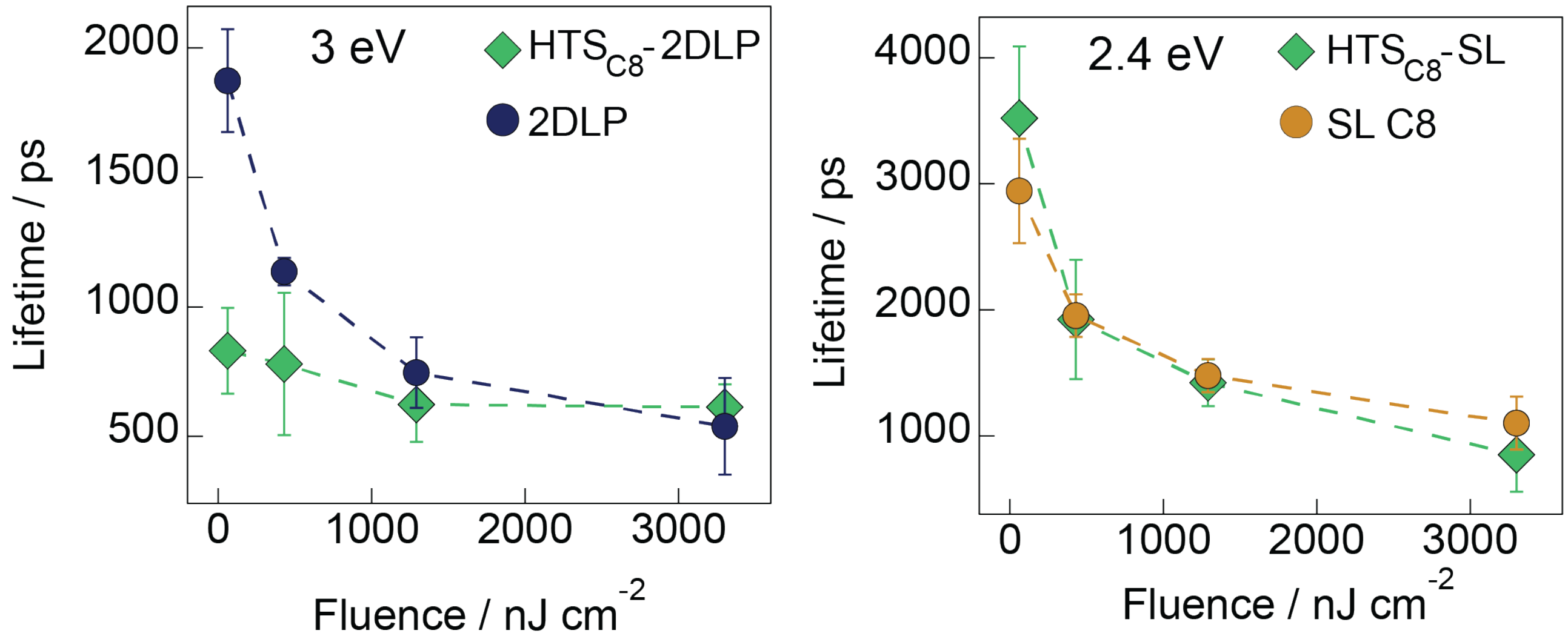

Figure S18: Effective lifetimes extracted at room temperature at 3.0 eV and 2.4 eV emission energies, respectively. For fluences above 60 $\mathrm{nJ\,cm^{-2}}$, the effective lifetime was calculated as the amplitude-weighted average of the two decay components obtained from the biexponential fits performed using equation S4. Each data point represents the mean of three independent measurements, and the error bars indicate the standard deviation.

S10. CRYOGENIC OPTICAL CHARACTERIZATION

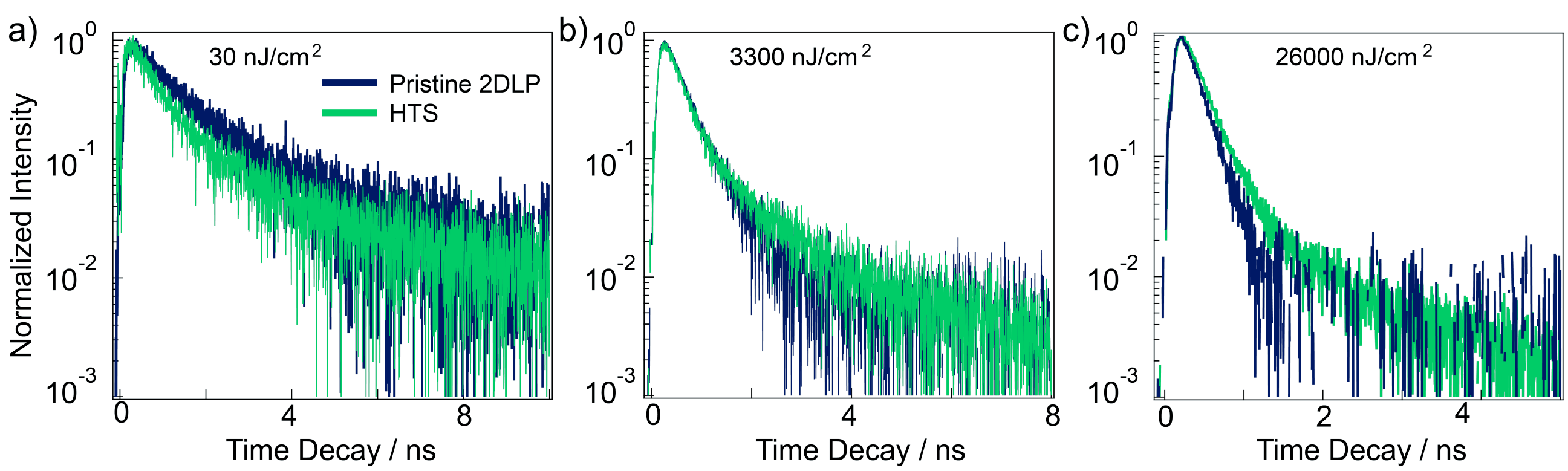

Figure S19: a-c) Time resolved photoluminescence decays measured for pristine 2DLP microcrystals at 80 K.

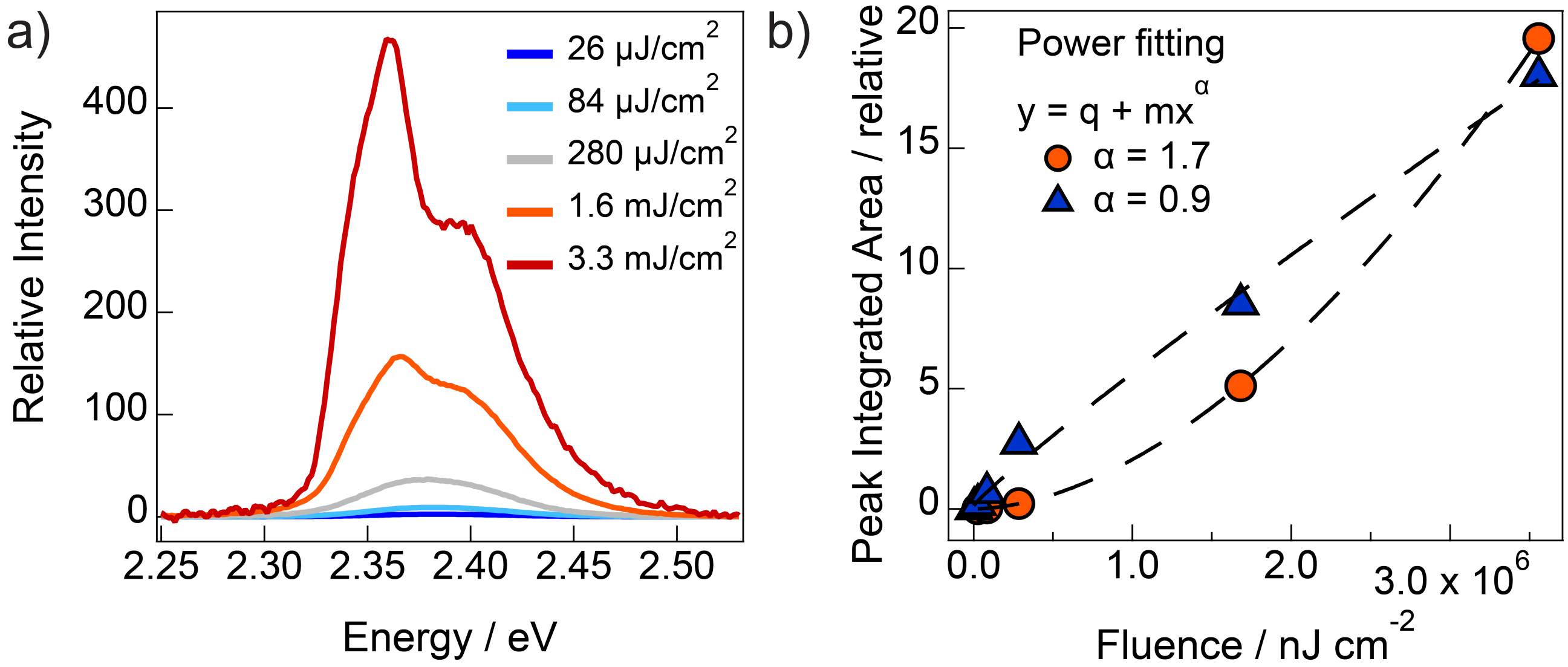

Figure S20: a) Photoluminescence of $CsPbBr_3$ pristine superlattice measured at 80 K as a function of fluence. b) Integrated area of high energy (blue triangles) and low energy (red circles) peaks as a function of fluence. Dashed lines represent the power law fitting.

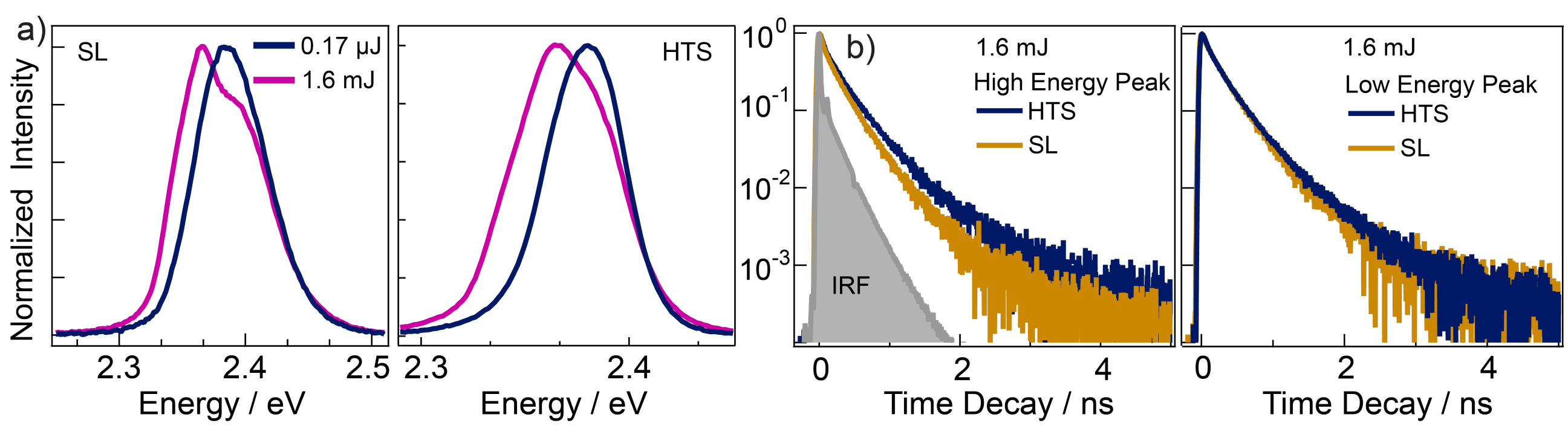

Figure S21: a) Comparison of the emission spectra in the low (170 nJ/cm^2) and high (1.6 mJ/cm^2) fluence regimes for a pristine superlattice (left graph) and a superlattice assembled in a heterostructure b) Time-resolved emission decays of pristine superlattice and heterostructure extracted for the high (left) and low (right) energy emission peaks.

[1] F. Di Stasio, S. Christodoulou, N. Huo, and G. Konstantatos, Near-unity photoluminescence quantum yield in cspbbr3 nanocrystal solid-state films via postsynthesis treatment with lead bromide, Chemistry of Materials **29**, 7663 (2017).

[2] S. Toso, D. Baranov, D. Altamura, F. Scattarella, J. Dahl, X. Wang, S. Marras, A. P. Alivisatos, A. Singer, C. Giannini, *et al.*, Multilayer diffraction reveals that colloidal superlattices approach the structural perfection of single crystals, ACS Nano **15**, 6243 (2021).

[3] S. Toso, D. Baranov, U. Filippi, C. Giannini, and L. Manna, Collective diffraction effects in perovskite nanocrystal superlattices, Accounts of Chemical Research **56**, 66 (2022).

[4] U. Filippi, S. Toso, M. L. Zaffalon, A. Pianetti, Z. Li, S. Marras, L. Goldoni, F. Meinardi, S. Brovelli, D. Baranov, *et al.*, Cooling-induced order–disorder phase transition in $CsPbBr_3$ nanocrystal superlattices, Advanced Materials **37**, 2410949 (2025).

[5] L. G. Feld, S. C. Boehme, V. Morad, Y. Sahin, C. J. Kaul, D. N. Dirin, G. Rainò, and M. V. Kovalenko, Quantifying forster resonance energy transfer from single perovskite quantum dots to organic dyes, ACS Nano **18**, 9997 (2024).

[6] M. Zhao, Y. Shi, J. Dai, and J. Lian, Ellipsometric study of the complex optical constants of a $CsPbBr_3$ perovskite thin film, Journal of Materials Chemistry C **6**, 10450 (2018).

[7] Z. Chu, H. Chen, X. Mao, Y. Li, W. Xu, and G. Ran, Anisotropic exciton–polaritons in 2D single-crystalline PEA_2PbBr_4 perovskites at room temperature, Journal of Physics D: Applied Physics **56**, 105301 (2023).

[8] J. Hardy, M. W. Brett, A. Rossi, I. Wagner, K. Chen, M. S. Timmer, B. L. Stocker, M. B. Price, and N. J. Davis, Energy transfer between anthracene-9-carboxylic acid ligands and $CsPbBr_3$ and $CsPbI_3$ nanocrystals, The Journal of Physical Chemistry C **125**, 1447 (2021).

[9] T. Yamaguchi, T. Fukumasu, Y. Ishibashi, and S. Masuo, Energy transfer from multiple excitons in a perovskite nanocrystal to organic dyes revealed by single-particle spectroscopy, Journal of the American Chemical Society **147**, 33944 (2025).

[10] B. W. van der Meer, Förster theory, FRET–Förster Resonance Energy Transfer , 23 (2013).
